# SNORD60-mediated 2′-O-methylation of KCP enhances ferroptosis sensitivity in hepatoblastoma

**DOI:** 10.1038/s41420-026-03160-5

**Published:** 2026-05-22

**Authors:** Jiabei Zhu, Wenxuan Ni, Zhixuan Bian, Yixuan Xiao, Jing Xiao, Hongwen Zhu, Ni Zhen, Qi Zhang, Cizhong Jiang, Ji Ma, Fenyong Sun, Qiuhui Pan

**Affiliations:** 1 Department of Clinical Laboratory, Shanghai Children’s Medical Center, Shanghai Jiao Tong University School of Medicine, Shanghai, China; 2 Department of Laboratory Medicine, Shanghai Tenth People’s Hospital of Tongji University, Shanghai, China; 3 Shanghai Tenth People’s Hospital, School of Life Sciences and Technology, Tongji University, Shanghai, China; 4 Precise Genome Engineering Center, School of Life Sciences, Guangzhou University, Guangzhou, China; 5 Faculty of Medical Laboratory Science, College of Health Science and Technology, Shanghai Jiao Tong University School of Medicine, Shanghai, China; 6 Shanghai Key Laboratory of Clinical Molecular Diagnostics for Pediatrics, Shanghai, China; 7 Hainan Branch, Shanghai Children’s Medical Center, School of Medicine, Shanghai Jiao Tong University, Sanya, China

**Keywords:** Oncogenesis, Paediatric cancer

## Abstract

Ferroptosis has increasingly emerged as a novel target for cancer therapy because of intrinsic or acquired ferroptosis vulnerabilities in cancers. Small nucleolar RNA (snoRNA)-mediated mRNA modifications play a critical role in regulating ferroptosis and supporting tumor cell adaptation. However, the function and mechanism of the box C/D snoRNA SNORD60 in hepatoblastoma (HB) remain poorly understood. Here, we found that SNORD60 expression was markedly downregulated in HB tissues, and restoring its expression inhibited HB cell proliferation and induced ferroptosis both in vitro and in vivo. Mechanistically, SNORD60 guided the 2′-O-methylation (Nm) of kielin/chordin-like protein (KCP) mRNA, and accelerated its degradation by resolving a G-quadruplex proximal to the Nm site. Furthermore, Silencing KCP induced lipid peroxidation and ferroptosis by suppressing ATF4-mediated transcription of SLC7A11. Moreover, plasma KCP protein levels were markedly higher in patients with HB than in healthy individuals, supporting KCP as a reliable non-invasive diagnostic biomarker for HB. High KCP expression correlates with poor patient prognosis. Overall, our findings reveal that SNORD60-mediated Nm modification of KCP prevents tumor cells from evading ferroptosis and inhibits tumor progression. This SNORD60/KCP axis represents a novel ferroptosis vulnerability and a promising therapeutic target in HB.

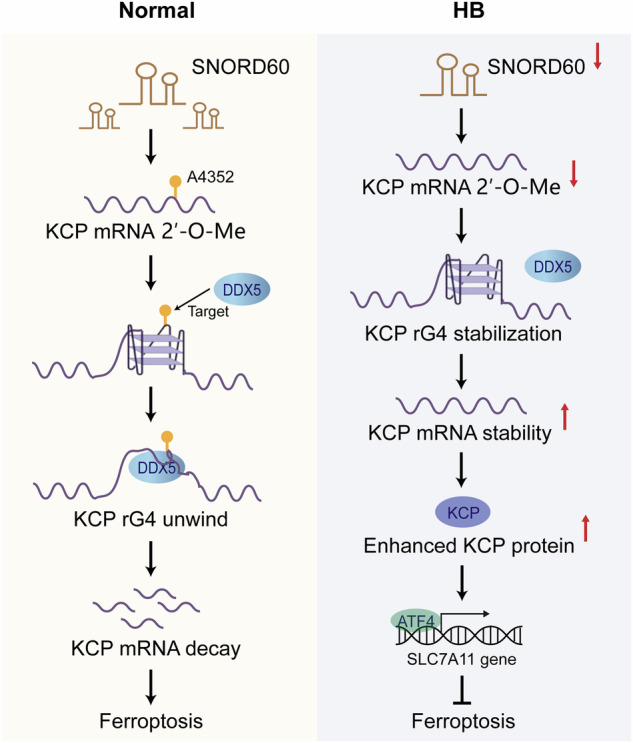

## Introduction

Hepatoblastoma (HB) is a malignant liver tumor that arises from dysdifferentiated hepatoblasts and accounts for approximately 70% of primary liver tumors in children under five years old [1]. In China, the incidence of HB has been steadily increasing in recent years, reaching 1.5 to 2 cases per million children [2]. Advances in hepatectomy and neoadjuvant chemotherapy have improved the 5-year survival rate for early-stage patients to about 70% [3]. However, the insidious onset and delayed diagnosis of HB often hinder surgical intervention in advanced-stage cases [4]. Furthermore, first-line chemotherapy is frequently ineffective in high-risk and refractory patients. Our previous studies have shown that serum α-fetoprotein (AFP), though widely used, lacks sufficient specificity and diagnostic accuracy, limiting its reliability as a clinical biomarker for HB [5]. Additionally, HB typically exhibits a relatively simple mutational landscape, with a low mutation burden (approximately 3.5 mutations per Mb) [6]. These challenges highlight the urgent need to investigate the molecular mechanisms underlying HB pathogenesis beyond genetic mutations and to identify novel biomarkers and therapeutic targets, particularly for high-risk patients.

Ferroptosis is a novel form of programmed cell death characterized by iron (Fe²⁺)-mediated oxidation of polyunsaturated fatty acids on cell membranes [7]. The cystine/glutamate antiporter system x_C_^–^, composed of the SLC3A2 and SLC7A11 heterodimer, plays a key role in regulating ferroptosis. Direct inhibition of system x_C_^–^ by erastin disrupts cystine uptake and glutamate efflux, leading to reduced glutathione synthesis and accumulation of lipid peroxides, ultimately triggering ferroptotic cell death [8]. Cancer cells often upregulate amino acid transporters and reprogram amino acid metabolism, which supports their survival and proliferation [9]. Among these, SLC7A11 has been reported to be upregulated in liver tumor tissues of HB patients, and endows resistance to ferroptosis [10]. Therefore, inducing ferroptosis is a promising therapeutic strategy for HB and may help overcome the limitations of conventional chemotherapy, particularly in tumors that evade other forms of programmed cell death.

Post-transcriptional chemical modifications of RNA species play crucial roles in regulating RNA metabolism and function, thereby influencing gene expression. Among these, 2’-O-methylation (Nm) is one of the most prevalent RNA modifications in eukaryotes. It occurs extensively on the ribose 2’-hydroxyl group of all four ribonucleosides, particularly in ribosomal RNA (rRNA) and transfer RNA (tRNA). These modifications, typically guided by C/D box small nucleolar RNAs (snoRNAs), are enriched in conserved functional regions of rRNA and impact various biological processes, including rRNA precursor maturation and protein synthesis [11–13]. Defects in snoRNA-mediated rRNA modifications have been shown to play critical roles in cancer initiation and progression. In our previous study, we demonstrated that abnormal intron retention in lncRNA-SNHG19, the host gene of SNORD60, significantly reduces SNORD60 production in HB cells [14], suggesting that SNORD60 may be transcriptionally dysregulated in HB. Interestingly, SNORD60 appears to have minimal impact on 28S rRNA maturation, indicating that it may function through non-canonical regulatory mechanisms. However, the biological role of SNORD60 and the molecular mechanisms underlying its function in HB remain largely unknown. Recent advances in high-throughput sequencing technologies have enabled the mapping of Nm sites at single-nucleotide resolution, revealing that Nm modifications also occur in low-abundance RNA species such as cellular mRNAs [15]. However, the characteristics and functional implications of mRNA Nm modifications remain poorly understood.

In this study, we found that SNORD60 expression was significantly lower in HB tissues compared with matched non-tumor liver tissues. Functional assays showed that overexpression of SNORD60 suppressed HB cell proliferation and inhibited orthotopic tumor growth in HB models. Mechanistically, SNORD60 directly bound to Kielin/chordin-like protein (KCP) mRNA and guided its 2’-O-methylation (Nm), thereby promoting KCP mRNA destabilization. In HB cells, SNORD60 depletion upregulated KCP, which in turn enhanced ATF4-dependent transcription of SLC7A11, resulting in reduced lipid peroxidation and increased resistance to ferroptosis. Furthermore, plasma KCP protein levels were markedly elevated in HB patients compared with healthy controls, and high KCP expression was associated with poor prognosis. These findings highlight KCP as a promising diagnostic and prognostic biomarker for HB and suggest that targeting the SNORD60/KCP/SLC7A11 axis to enhance ferroptosis sensitivity may represent a potential therapeutic strategy for HB patients.

## Results

### Downregulation of SNORD60 endows HB cells with resistance to ferroptosis

**Fig. 1:**
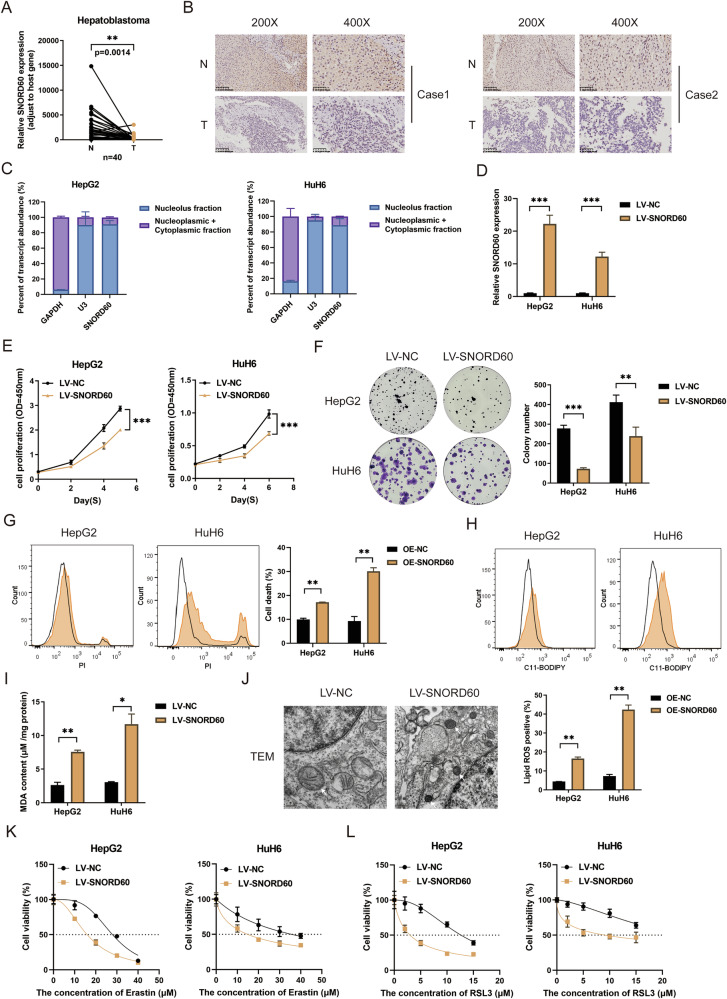
Significant downregulation of SNORD60 promotes cell proliferation and inhibits ferroptosis in HB. **A** Relative expression level of SNORD60 in 40 paired HB and adjacent non-tumor liver tissues were measured by qRT‒PCR. **B** Representative ISH images showing SNORD60 staining in 2 pairs of HB and non-tumor liver tissues. **C** SNORD60 expression level in subcellular fractions was measured by qRT‒PCR. **D** Relative expression level of SNORD60 in HB/LV-NC and HB/LV-SNORD60 cells were measured by qRT‒PCR. **E**, **F** Detection of proliferative activity of HB/LV-NC and HB/LV-SNORD60 cells by CCK-8 assay (**E**) and colony formation assay (**F**). **G**, **H** Quantitation of cell death (**G**) and lipid ROS levels (**H**) by PI and C11-BODIPY staining, respectively, coupled with flow cytometry in HB cells transfected with OE-NC or OE-SNORD60 plasmids. **I** Determination of MDA content in HB/LV-NC and HB/LV-SNORD60 cells. **J** Transmission electron microscopy images of HepG2/LV-NC and HepG2/LV-SNORD60 cells. White arrowheads indicate shrunken mitochondria in SNORD60-overexpressing HB cells. **K**, **L** Determination of the viability of HB/LV-NC and HB/LV-SNORD60 cells after treatment with the indicated concentrations of erastin (**K**) and RSL3 (**L**).

To investigate the differential expression of snoRNAs in HB, we previously performed snoRNA sequencing on four pairs of HB tissues and matched adjacent non-tumor liver tissues [16]. This analysis identified 72 significantly dysregulated snoRNAs, including 8 upregulated and 64 downregulated snoRNAs (Fig. S1A). Notably, 70.31% of these dysregulated snoRNAs belonged to the box C/D family, suggesting that this class of snoRNAs may play an important role in HB pathogenesis. Among these box C/D snoRNA candidates, SNORD60 was one of the most significantly dysregulated snoRNAs and was selected for further investigation. Our previous work revealed that abnormal intron retention of its host gene, the oncogenic lncRNA-SNHG19, leads to reduced SNORD60 production in HB cell lines [14]. These transcriptomic findings suggested that SNORD60 may function as a regulatory snoRNA involved in HB tumorigenesis. Therefore, we assessed SNORD60 expression in 40 paired HB tissues and adjacent non-tumor liver tissues using qRT‒PCR. The results revealed a significant reduction in SNORD60 expression in HB tissues compared to non-tumor tissues (Fig. 1A). ISH of an HB tissue microarray further confirmed that SNORD60 staining intensity was lower in HB tissues than in non-tumor liver tissues (Fig. 1B). Subcellular fractionation of HepG2 and HuH6 cells demonstrated that SNORD60 was predominantly localized in the nucleolus (Fig. 1C). To investigate the role of SNORD60 in HB, we established HB cell lines stably overexpressing SNORD60 and confirmed a 12.2–22.3-fold increase in its expression by qRT‒PCR (Fig. 1D). CCK-8 and colony formation assays showed that SNORD60 overexpression significantly inhibited HB cell proliferation (Fig. 1E, F). Propidium iodide (PI) staining indicated that SNORD60 overexpression induced much more potent cell death (Fig. 1G). However, it did not significantly stimulate apoptotic cell death (Fig. S1B, C). To explore the molecular mechanism by which SNORD60 regulates HB cell death, RNA sequencing was performed in HepG2/LV-NC and HepG2/LV-SNORD60 cells. This analysis identified 884 significantly differentially expressed mRNAs, including 426 upregulated and 458 downregulated transcripts (Fig. S1D and Table S2). Functional enrichment analysis revealed that the ferroptosis pathway was among the top 20 enriched KEGG pathways (Fig. S1E), suggesting a potential role of SNORD60 in regulating ferroptotic cell death.

Ferroptosis is a regulated form of cell death primarily driven by disruption of cellular antioxidant defenses, particularly the system xc⁻–glutathione (GSH)–glutathione peroxidase 4 (GPX4) axis, leading to the accumulation of lipid hydroperoxides [17]. To further investigate whether SNORD60 regulates ferroptosis in HB, we performed flow cytometry using the fluorescent probe C11-BODIPY and malondialdehyde (MDA) content measurement. These analyses revealed significantly increased levels of lipid reactive oxygen species (ROS) in SNORD60-overexpressing cells (Fig. 1H, I). Increased cellular labile iron content promotes ferroptosis by directly oxidizing lipids through the Fenton reaction and by acting as a cofactor for lipid-oxidizing enzymes [18]. Therefore, we measured the Fe^3+^/Fe^2+^ and reduced/oxidized glutathione (GSH/GSSG) ratios to evaluate the role of SNORD60 in ferroptosis regulation. The results showed a marked reduction in the Fe^3+^/Fe^2+^ ratio in SNORD60-overexpressing cells (Fig. S1F). Consistently, the GSH/GSSG ratio was also decreased upon SNORD60 overexpression (Fig. S1G), indicating enhanced lipid peroxidation in the cellular microenvironment. Transmission electron microscopy showed that these cells exhibited characteristic morphologic features of ferroptosis, including shrunken mitochondria with increased membrane density (Fig. 1J). Furthermore, SNORD60 overexpression enhanced iron-dependent oxidative cell death induced by the system x_C_^–^ inhibitor erastin and the GPX4 inhibitor RSL3 (Fig. 1K, L).

**Fig. 2:**
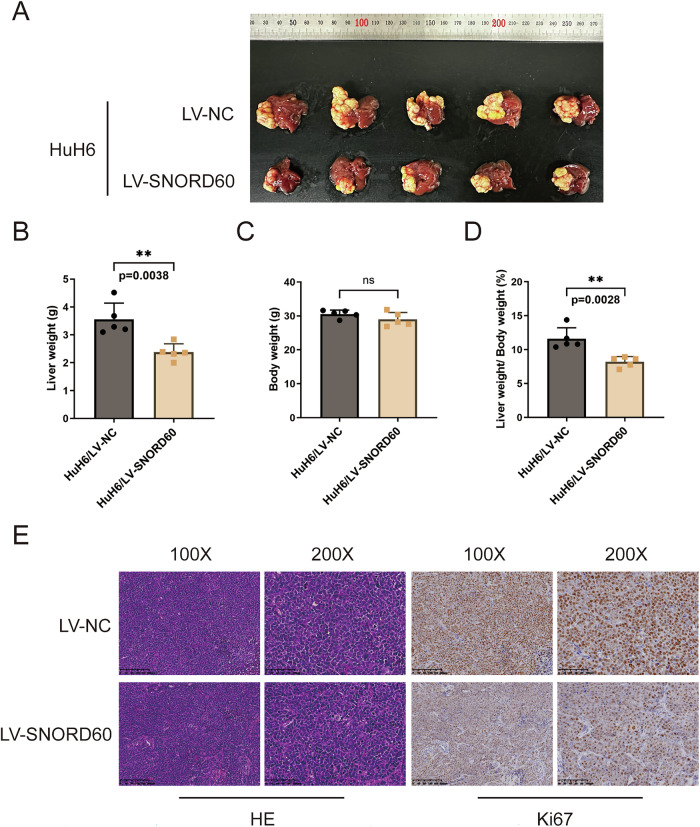
Overexpression of SNORD60 inhibits HB tumor growth in vivo. **A** The livers dissected from ten nude mice orthotopically inoculated with HuH6/LV-NC and HuH6/LV-SNORD60 cells. **B**–**D** Average liver weight (**B**), average body weight (**C**), and liver-to-body weight ratio (**D**) of nude mice were calculated. **E** Representative IHC images of HE (left) and Ki67 staining (right) in dissected liver tissues.

To evaluate whether SNORD60 inhibits tumor growth in vivo, we employed an orthotopic xenograft liver tumor model (Fig. 2A). Tumors in the SNORD60-overexpressing group were significantly smaller, and liver weights were markedly lower than those in the control group (Fig. 2B–D). IHC analysis revealed that the staining intensities of Ki67-positive cells was significantly lower in the SNORD60-overexpressing tumors compared to controls (Fig. 2E). Collectively, these findings identify the tumor-suppressive SNORD60 as a novel negative regulator of ferroptosis in HB cells.

### SNORD60-mediated 2’-O-methylation (Nm) at KCP A4352 destabilizes KCP mRNA

**Fig. 3:**
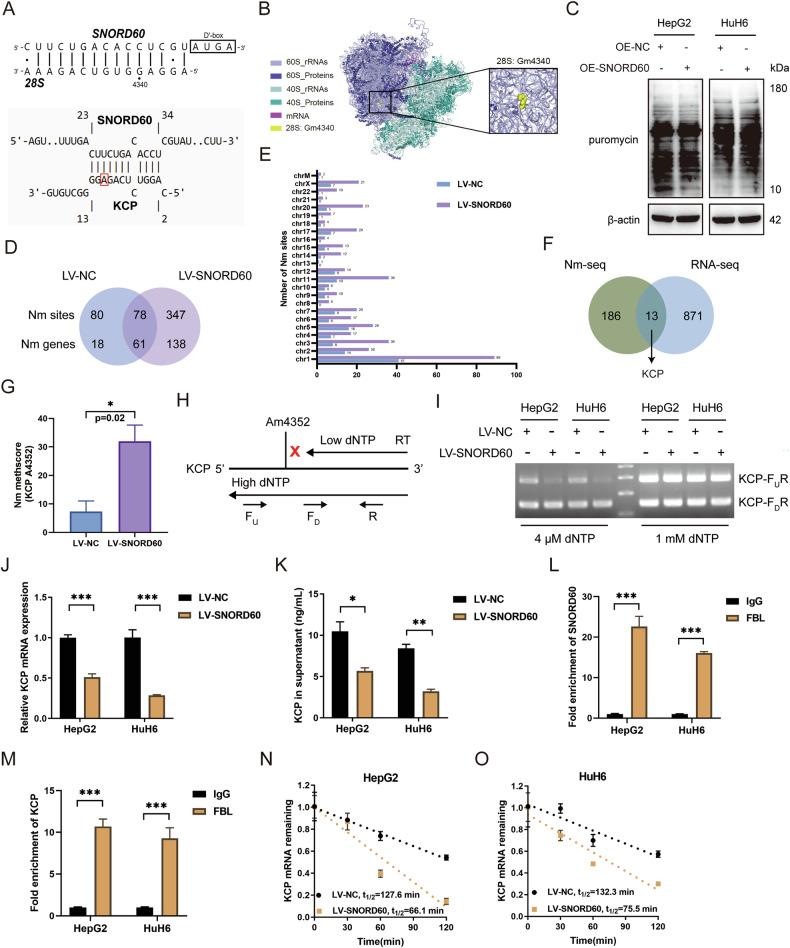
SNORD60 guides the 2’-O-methylation (Nm) of KCP mRNA to promote its decay. **A** Predicted interaction motifs between SNORD60 and 28S rRNA (top) and between SNORD60 and KCP mRNA (bottom). The G4340 site of 28S rRNA and the A4352 site of KCP, the Nm targets of SNORD60, are indicated in the red boxes. **B** PyMOL analysis of the eukaryotic ribosome showing the SNORD60 methylation site G4340 within the 28S ribosomal subunit. **C** Global protein synthesis in HB/OE-NC and HB/OE-SNORD60 cells after treatment with 20 μg/ml puromycin were measured by Western blotting assays. **D** Venn diagram illustrating the number of Nm sites and Nm genes identified by Nm-seq in HepG2/LV-NC and HepG2/LV-SNORD60 cells. **E** Distribution of Nm sites across different chromosomes. **F** Venn diagram illustrating the number of shared significantly differentially Nm-modified genes from Nm-seq and RNA-seq analyses of HepG2/LV-NC and HepG2/LV-SNORD60 cells. **G** The methscore of KCP A4352 site from Nm-seq in HepG2/LV-NC and HepG2/LV-SNORD60 cells. **H** Schematic diagram of RTL-PCR method for detecting Nm modification at the KCP A4352 site. **I** KCP Nm modification activity at the A4352 site in HB/LV-NC and HB/LV-SNORD60 cells was detected by RTL-PCR assay. (**J**, **K**) The mRNA (**J**) and protein (**K**) levels of KCP in HB/LV-NC and HB/LV-SNORD60 cells were measured by qRT‒PCR and ELISA assays. **L**, **M** RIP assays with anti-FBL antibody followed by RT-qPCR to detect the enrichment of SNORD60 (**L**) and KCP mRNA (**M**). **N**, **O** Relative mRNA level of KCP in HB/LV-NC and HB/LV-SNORD60 cells was measured using qRT‒PCR after treatment with 5 μg/ml actinomycin D for the indicated times.

Aberrant expression of box C/D snoRNAs disrupts 2′-O-methylation (Nm) of canonical rRNA targets, impairing ribosome function and protein synthesis, thereby promoting cancer progression [19]. SNORD60 is predicted to guide Nm modification at position G4340 of 28S rRNA (Fig. 3A); however, our previous study demonstrated that it has only a minimal impact on the maturation of 28S rRNA precursor [14]. Structural analysis using PyMol revealed that G4340 is located relatively far from the ribosomal decoding center in mature ribosomes (Fig. 3B), suggesting that its modification may have limited influence on overall translational activity. Consistent with this, puromycin incorporation assays showed that SNORD60 overexpression had only a minor effect on global protein translation efficiency in HB cells (Fig. 3C).

To identify potential mRNA targets underlying the pro-ferroptotic role of SNORD60, we performed Nm-seq in HB cells. In the control group, 158 Nm sites were detected in 79 RefSeq-annotated genes, whereas the SNORD60-overexpressing group exhibited 425 Nm sites across 199 genes (Fig. 3D and Table S2). Although Nm sites were distributed in the vast majority of chromosomes, chromosome 1 showed a significantly higher number of methylation events than expected by chance (Fig. 3E). To further investigate functionally relevant targets, we conducted transcriptomic analyses and identified 13 differentially expressed genes that overlapped with Nm-modified genes upon SNORD60 overexpression (Fig. 3F and Tables S2, 3). We next used IntaRNA tool to predict potential interactions between these candidates and SNORD60. Among them, KCP emerged as a key target. This analysis revealed complementary base pairing between SNORD60 and the sequence adjacent to the A4352 site of KCP (Fig. 3A). Notably, the SNORD60-binding sequence in KCP was identical to that in 28S rRNA, consistent with the known mechanism of box C/D snoRNAs, which rely on direct sequence complementarity to guide methylation.

Nm-seq and RTL-P assay confirmed that Nm modification at the KCP A4352 site was significantly enhanced in SNORD60-overexpressing cells (Fig. 3G–I). However, both the mRNA and protein levels of KCP were markedly reduced upon SNORD60 overexpression, as determined by qRT‒PCR and Western blotting (Fig. 3J, K). Nm modification is typically catalyzed by fibrillarin (FBL), a core component of the small nucleolar ribonucleoprotein (snoRNP) complex, which is recruited to target RNAs by specific box C/D snoRNAs [20]. RIP assays demonstrated that both SNORD60 and KCP mRNA bound to FBL (Fig. 3L, M), suggesting that SNORD60 guides FBL-dependent Nm modification of KCP mRNA at the A4352 site. To explore the mechanism by which SNORD60 downregulates KCP, we performed RNA decay assays and found that the half-life of KCP mRNA was significantly reduced in SNORD60-overexpressing cells compared to controls (Fig. 3N–O). These results indicate that SNORD60-guided Nm modification at KCP A4352 promotes its mRNA degradation.

### Nm recruits DDX5 helicase to resolve the G-quadruplex structure of KCP mRNA

**Fig. 4:**
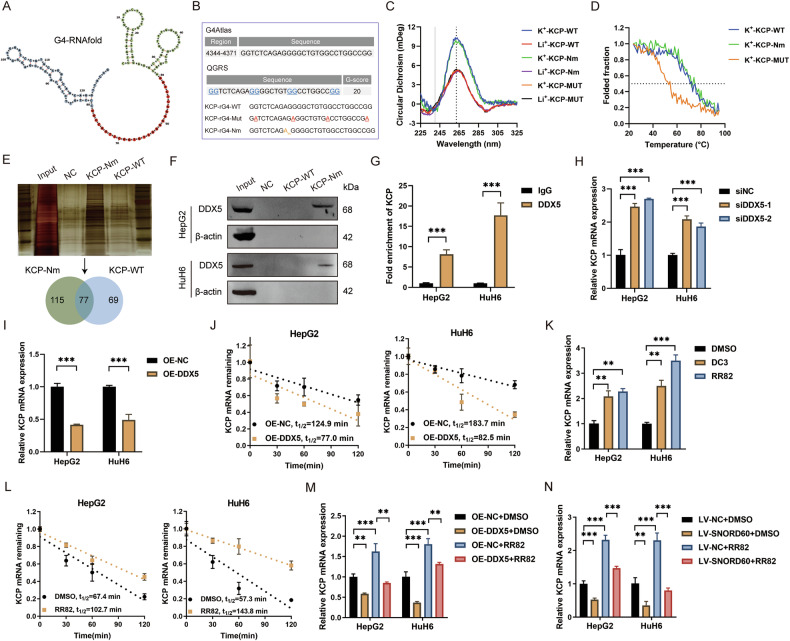
Nm modification of KCP recruits DDX5 to resolve the adjacent rG4 structure which maintains KCP mRNA stability. **A**, **B** Putative rG4-forming sequence in KCP mRNA predicted using the G4Atlas and QGRS mapper. **C** The circular dichroism (CD) spectra of wild-type KCP (KCP-WT), Nm-modified KCP (KCP-Nm), and mutant KCP (KCP-MUT) under KCl or LiCl conditions. **D** Thermal denaturing profiles of KCP-WT, KCP-Nm and KCP-MUT under KCl conditions detected by monitoring CD signals at 262 nm. **E** RNA pull-down assays using wild-type or Nm-modified KCP mRNA probes followed by label-free quantitative proteomics to identify proteins binding KCP mRNA. **F** RNA pull-down assays using wild-type or Nm-modified KCP mRNA probes followed by Western blotting to detect the interaction between DDX5 protein and KCP mRNA. **G** RIP assays with anti-DDX5 antibody followed by RT-qPCR to detect KCP mRNA enrichment. **H** Relative mRNA level of KCP in HB cells transfected with siRNAs targeting DDX5 were measured by qRT‒PCR. **I** Relative mRNA level of KCP in HB cells transfected with OE-NC or OE-DDX5 plasmids were measured by qRT‒PCR. **J** Relative mRNA level of KCP in HB/OE-NC and HB/OE-DDX5 cells was measured by qRT‒PCR after treatment with 5 μg/ml actinomycin D for the indicated times. **K** Relative mRNA level of KCP in HB cells was measured by qRT‒PCR after treatment with DC3 or RR82. **L** Relative mRNA level of KCP in HB cells was measured by qRT‒PCR after treatment with RR82 followed by 5 μg/ml actinomycin D for the indicated times. **M** Relative mRNA level of KCP in HB/OE-NC and HB/OE-DDX5 cells was measured by qRT‒PCR after treatment with RR82. **N** Relative mRNA level of KCP in HB/LV-NC and HB/LV-SNORD60 cells was measured by qRT‒PCR after treatment with RR82.

To further investigate how SNORD60-mediated Nm modification contributes to the destabilization of KCP mRNA, we analyzed the secondary structure surrounding the Nm site using the G4Atlas and QGRS databases. This analysis revealed that the A4352 site is located adjacent to a potential RNA G-quadruplex (rG4) sequence (Fig. 4A, B). To evaluate the structural characteristics of this rG4 motif in different contexts—wild-type, 2’-O-methylated, or mutated—we synthesized a series of RNA oligomers: KCP-rG4-WT, KCP-rG4-Nm, and KCP-rG4-Mut. Circular dichroism (CD) spectroscopy and thermal stability assays were then performed on these oligomers under two conditions: 150 mM KCl (G4-stabilizing) and 150 mM Li+ (G4-non-stabilizing). CD spectra of KCP-rG4-WT and KCP-rG4-Nm exhibited a characteristic positive peak at ~262–265 nm and a negative peak at ~240–245 nm under K+ conditions. In contrast, the KCP-rG4-Mut showed much weaker signals, suggesting disrupted G4 formation (Fig. 4C). The CD spectrum of KCP-rG4-WT were significantly stronger in K+ compared to Li + , confirming the formation of parallel G-quadruplex structures in the presence of potassium. Thermal melting analysis showed that both the wild-type and 2’-O-methylated KCP rG4 motifs formed stable rG4 structures, which were highly thermostable (Fig. 4D).

To uncover the regulatory mechanism by which Nm modification and rG4 structure affect KCP mRNA, we performed RNA pull-down assays followed by label-free quantitative proteomics to identify proteins interacting with the KCP-rG4-WT or KCP-rG4-Nm motifs (Fig. 4E and Table S4). DEAD-box helicase 5 (DDX5), known for resolving DNA and RNA G4s [21, 22], was found to specifically bind to the KCP-rG4-Nm motif but not the wild-type, as validated by RNA pull-down followed by Western blotting, and RIP assays(Fig. 4F–G). Functionally, silencing DDX5 in HB cells increased KCP mRNA levels, whereas DDX5 overexpression reduced them (Fig. 4H–I and Fig. S2A–B). RNA decay assays demonstrated that KCP mRNA had a shorter half-life in DDX5-overexpressing cells compared to controls (Fig. 4J), indicating that DDX5 promotes KCP mRNA decay by unwinding the rG4 structure.

To test this hypothesis, we treated cells with two G4-stabilizing compounds, PhenDC3 and RR82. Both treatments increased KCP mRNA levels and prolonged its half-life (Fig. 4K–L), without affecting DDX5 mRNA expression (Fig. S2C). Furthermore, RR82 blocked the DDX5-induced downregulation of KCP mRNA (Fig. 4M) and reversed the KCP mRNA reduction caused by SNORD60 overexpression (Fig. 4N). To further confirm the involvement of the DDX5/KCP axis in SNORD60-mediated effects, we transfected DDX5 siRNAs into HB cells overexpressing SNORD60. DDX5 depletion rescued the SNORD60-induced reduction in KCP mRNA levels (Fig. S2D). Functionally, silencing DDX5 also reversed the SNORD60-mediated inhibition of cell proliferation and colony formation (Fig. S2E–H). Collectively, these results strongly suggest that SNORD60-mediated Nm at the A4352 site of KCP mRNA destabilizes the transcript by recruiting DDX5 to unwind its rG4 structure.

### Silencing KCP inhibits cell proliferation and induces ferroptosis in HB

**Fig. 5:**
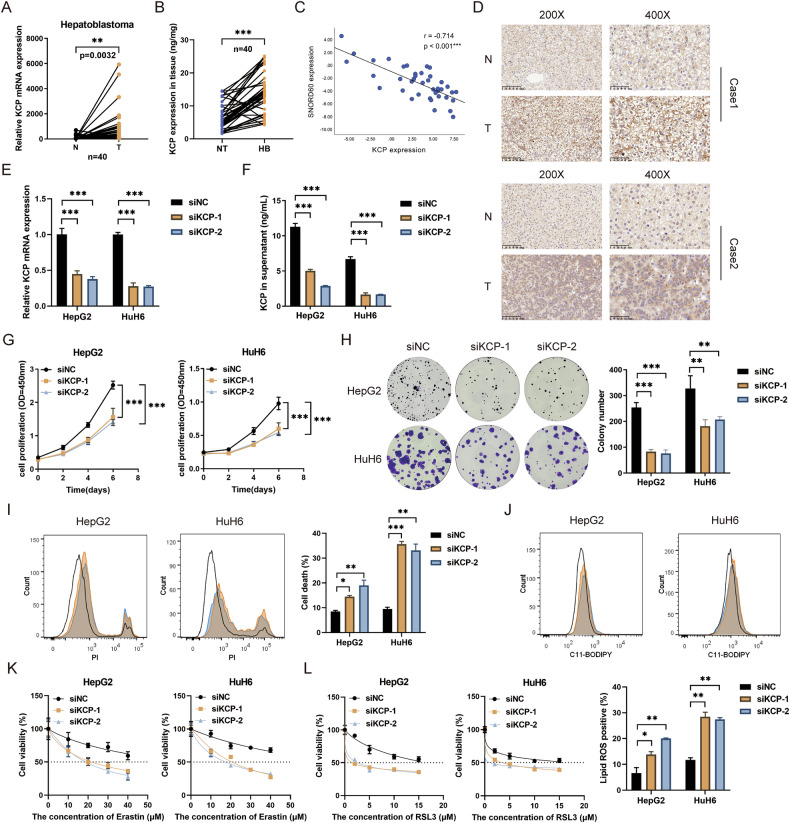
Knockdown of KCP inhibits cell proliferation and promotes ferroptosis in HB. **A** Relative mRNA level of KCP in 40 paired HB and adjacent non-tumor liver tissues were measured by qRT‒PCR. **B** The protein level of KCP in 40 paired HB and adjacent non-tumor liver tissues were measured by ELISA. **C** Spearman rank correlation analysis of SNORD60 and KCP expression levels. **D** Representative IHC images of KCP staining in 2 paired HB and non-tumor liver tissues. **E**, **F** The mRNA (**E**) and protein (**F**) levels of KCP in HB cells transfected with siRNAs targeting KCP were measured by qRT‒PCR and ELISA assays. **G**, **H** Detection of cell proliferative activity by CCK-8 assay (**G**) and colony formation assay (**H**). Cells were subjected to the same treatment as in (**E**). **I**, **J** Quantitation of cell death (**I**) and lipid ROS levels (**J**) by PI and C11-BODIPY staining, respectively, coupled with flow cytometry. Cells were subjected to the same treatment as in (**E**). **K**, **L** Determination of the viability of HB cells transfected with siRNAs targeting KCP after treatment with the indicated concentrations of erastin (**K**) and RSL3 (**L**).

qRT‒PCR and ELISA assays confirmed a significant increase in KCP mRNA and protein levels in HB tissues compared to adjacent non-tumor liver tissues (Fig. 5A, B). Spearman’s rank correlation analysis revealed a strong negative correlation between SNORD60 and KCP mRNA expression levels (r = –0.714, ***P < 0.001) (Fig. 5C). IHC of HB tissue microarrays further demonstrated a higher proportion of HB tissues with strong KCP staining intensity compared to non-tumor liver tissues (Fig. 5D).

To explore the biological function of KCP in HB, we transfected HB cells with siRNAs targeting KCP or a negative control. Knockdown efficiency was validated by qRT‒PCR and ELISA (Fig. 5E, F). CCK-8 and colony formation assays showed that KCP silencing significantly suppressed HB cell proliferation (Fig. 5G, H). Moreover, KCP knockdown led to increased cell death and elevated lipid ROS levels, as assessed by PI staining and C11-BODIPY staining, respectively (Fig. 5I, J). Silencing KCP also enhanced ferroptotic cell death triggered by erastin and RSL3 (Fig. 5K, L). In contrast to the tumor-suppressive role of SNORD60, these results suggest that KCP promotes cell proliferation and suppresses ferroptosis in HB.

To further determine whether SNORD60 exerts its tumor-suppressive function through the downregulation of KCP, we co-transfected HB cells with SNORD60- and KCP-expressing plasmids. Overexpression of KCP reversed the SNORD60-induced reduction in KCP mRNA levels (Fig. S3A). Functionally, KCP restoration rescued the suppressed proliferation and colony-forming ability caused by SNORD60 overexpression (Fig. S3B–D). These findings collectively indicate that SNORD60 inhibits HB cell growth by downregulating KCP, further supporting the oncogenic role of KCP and the tumor-suppressive function of SNORD60.

### SNORD60-mediated KCP downregulation induces ferroptosis by inhibiting ATF4/SLC7A11 signaling

**Fig. 6:**
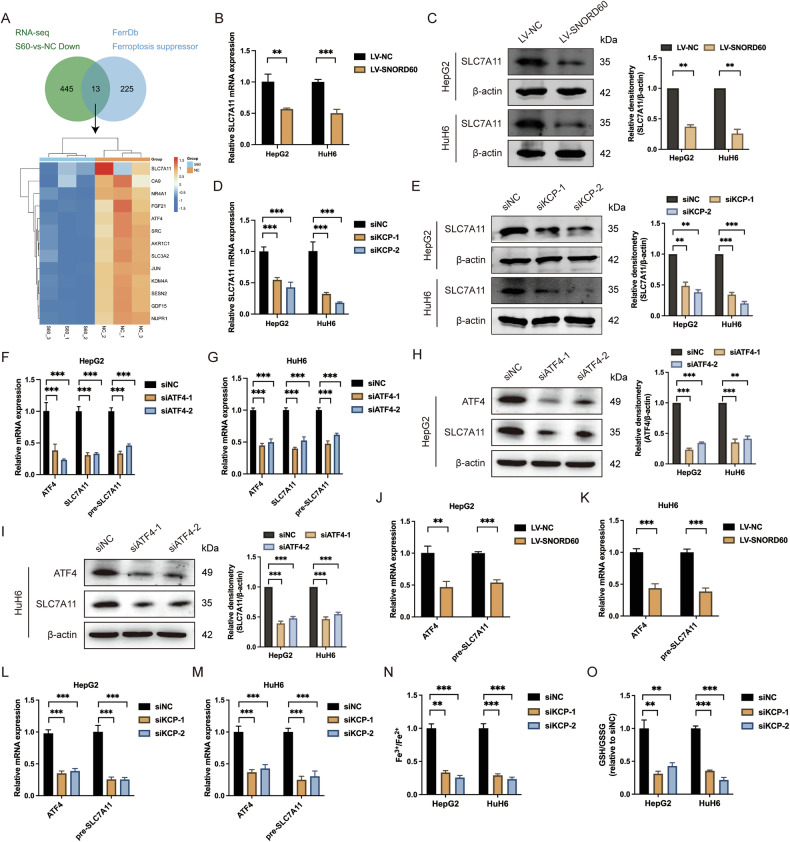
SNORD60/KCP axis promotes HB cell ferroptosis by inhibiting ATF4-mediated SLC7A11 transcription. **A** Venn diagram showing the intersectional targets between the downregulated genes in SNORD60-overexpressing cells and ferroptosis suppressors from FerrDb. Heatmap exhibiting downregulation of 13 ferroptosis suppressors in HepG2/LV-NC and HepG2/LV-SNORD60 cells. **B**, **C** The mRNA (**B**) and protein (**C**) levels of SLC7A11 in HB/LV-NC and HB/LV-SNORD60 cells were measured by qRT‒PCR and Western blotting assays. Relative densitometry was performed with ImageJ. **D**, **E** The mRNA (**D**) and protein (**E**) levels of SLC7A11 in HB cells silencing KCP were measured by qRT‒PCR and Western blotting assays. Relative densitometry was performed with ImageJ. **F**, **G** The mRNA levels of ATF4, pre-SLC7A11 and mature SLC7A11 in HB cells transfected with siRNAs targeting ATF4 were measured by qRT‒PCR assays. **H**, **I** The protein levels of ATF4 and SLC7A11 in HB cells transfected with siRNAs targeting ATF4 were measured by Western blotting assays. Relative densitometry was performed with ImageJ. **J**, **K** The mRNA levels of ATF4 and pre-SLC7A11 in HB/LV-NC and HB/LV-SNORD60 cells were measured by qRT‒PCR assays. **L**, **M** The mRNA levels of ATF4 and pre-SLC7A11 in HB cells silencing KCP were measured by qRT‒PCR assays. **N** The relative Fe^3+^/Fe^2+^ ratio in HB cells silencing KCP. **O** The relative GSH/GSSG ratio in HB cells silencing KCP.

To investigate the mechanism by which SNORD60-mediated downregulation of KCP promotes ferroptosis, we analyzed differentially expressed genes in SNORD60-overexpressing cells and compared them with well-known ferroptosis regulators from the FerrDb database. A total of 13 overlapping genes were identified between downregulated genes in SNORD60-overexpressing cells and established ferroptosis suppressors using a Venn diagram (Fig. 6A). Among these, we observed significant reductions in the expression of activating transcription factor 4 (ATF4) and its downstream target SLC7A11, the subunit of cystine/glutamate antiporter system xc⁻ that suppresses ferroptosis [18]. qRT‒PCR and Western blotting analyses confirmed that SNORD60 overexpression led to decreased levels of SLC7A11 mRNA and protein in HB cells (Fig. 6B, C). To determine whether this effect is mediated via KCP, we silenced KCP in HB cells and observed a notable reduction in both SLC7A11 mRNA (Fig. 6D) and protein levels (Fig. 6E), suggesting that KCP positively regulates SLC7A11 expression. Previous studies have shown that ATF4 protects against liver damage and tumor development by activating SLC7A11 transcription to inhibit stress-induced ferroptosis [23]. Consistently, we observed that ATF4 knockdown led to a marked reduction in precursor (pre-SLC7A11) and mature SLC7A11 transcripts (Fig. 6F, G), and SLC7A11 protein (Fig. 6H, I), indicating that ATF4 transcriptionally regulates SLC7A11. We next examined whether the SNORD60/KCP axis suppresses SLC7A11 transcription via ATF4. Both SNORD60 overexpression (Fig. 6J, K) and KCP knockdown (Fig. 6L, M) significantly decreased the mRNA levels of ATF4 and pre-SLC7A11, supporting the notion that SNORD60/KCP axis downregulates SLC7A11 by inhibiting ATF4-mediated transcription.

SLC7A11-mediated cystine uptake supports GSH biosynthesis and subsequent GPX4 activity, which protect cells from ferroptosis. In contrast, acyl-CoA synthetase long-chain family member 4 (ACSL4) promotes ferroptosis by facilitating the incorporation of polyunsaturated fatty acids into membrane phospholipids, thereby increasing susceptibility to lipid peroxidation [24]. To further evaluate the role of KCP in ferroptosis regulation, we measured the Fe^3^⁺/Fe^2^⁺ and GSH/GSSG ratios. The results showed that KCP knockdown led to a marked reduction in both the Fe^3^⁺/Fe^2^⁺ ratio (Fig. 6N) and the GSH/GSSG ratio (Fig. 6O), consistent with the effects observed following SNORD60 overexpression. Western blotting analyses further showed that SNORD60 overexpression decreased GPX4 protein level and increased ACSL4 expression in HB cells (Fig. S4A). Consistently, knockdown of KCP or ATF4 also decreased GPX4 levels while increasing ACSL4 expression in HB cells (Fig. S4B–C). Collectively, these findings indicate that SNORD60-mediated downregulation of KCP promotes ferroptosis by suppressing the ATF4/SLC7A11 signaling pathway, thereby weakening antioxidant defenses and enhancing lipid peroxidation.

### KCP is a potential diagnostic and prognostic biomarker for HB

**Fig. 7:**
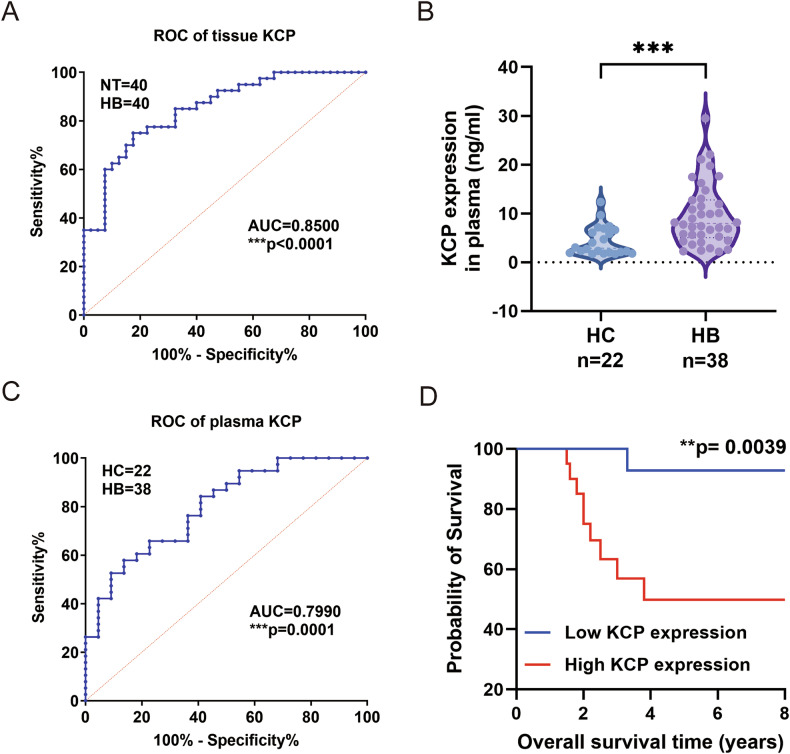
Plasma KCP is a potential diagnostic and prognostic biomarker in HB. **A** ROC curve analysis of tissue-derived KCP protein levels for HB diagnosis. **B** Plasma KCP protein concentrations in HB patients and healthy control were measured by ELISA assays. **C** ROC curve analysis of plasma-derived KCP protein levels for HB diagnosis. **D** Kaplan–Meier survival analysis of HB patients with low versus high KCP expression levels (mean cut-off). Statistical significance was assessed using the log rank test. Graphical abstract: Our findings suggest that SNORD60 promotes Nm modification of KCP mRNA and recruits DDX5 to resolve the adjacent rG4 structure of KCP, thereby destabilizing KCP mRNA. In HB cells, depletion of SNORD60 leads to aberrantly increased KCP expression, which activates ATF4-mediated SLC7A11 transcription, enhances resistance to ferroptosis, and ultimately promotes malignant progression of HB.

To evaluate the diagnostic value of KCP in HB, we performed receiver operating characteristic (ROC) curve analysis. KCP effectively distinguished 40 HB tissues from 40 adjacent non-tumor liver tissues, with an area under the curve (AUC) of 0.8500 (*P* < 0.0001) (Fig. 7A). We further evaluated KCP protein levels in plasma samples from healthy controls and HB patients. KCP levels were significantly elevated in the plasma of HB patients compared to healthy controls (Fig. 7B). ROC curve analysis revealed that plasma KCP could differentiate 38 HB patients from 22 healthy controls, with an AUC of 0.7990 (*P* = 0.0001) (Fig. 7C). To explore the clinical relevance of KCP, we used Fisher’s exact test to examine the association between KCP expression and clinicopathological characteristics of HB patients. The clinicopathological information of 40 HB patients is shown in Table S5. Based on ELISA results, patients were categorized into high and low KCP expression groups using the median expression value as the cutoff. High KCP expression was significantly associated with adverse clinical features, including advanced PRETEXT stage (*P* = 0.022), larger tumor size (*P* = 0.010), and metastasis (*P* = 0.031) (Table S6). Moreover, elevated KCP expression correlated with poorer overall survival in HB patients (Fig. 7D). Collectively, these findings suggest that KCP is a promising biomarker for both the diagnosis and prognosis of HB.

## Discussion

Although snoRNAs are well known for their essential roles in ribosome biogenesis and protein synthesis through the 2′-O-methylation (Nm) of canonical rRNA targets in their conserved functional regions [20], recent studies have revealed that they also regulate a variety of important biological processes—such as oncogene activation-mediated senescence, protein translocation, and secretion—by directly interacting with proteins or mRNAs [25, 26]. Accumulating evidence indicates that aberrant snoRNA expression contributes to the initiation and progression of multiple cancers [27, 28]. Our previous research demonstrated that SNORD60 expression is downregulated in HB cells due to abnormal intron retention in its host gene, snoRNA host gene 19 (SNHG19) [14]. In the present study, we show for the first time that SNORD60 suppresses cell proliferation and induces ferroptotic cell death in HB, acting independently of its canonical functional pathway. Like other classical box C/D snoRNAs, SNORD60 is predicted to guide the Nm modification at the G4340 site of 28S rRNA. Unexpectedly, our earlier work showed that SNORD60 has only a minor impact on 28S rRNA maturation [14]. Consistently, we observed no significant change in global protein translation efficiency upon SNORD60 overexpression in HB cells, suggesting a non-canonical mechanism of SNORD60-mediated biological process. To further investigate this, we performed Nm-seq to identify mRNAs modified by SNORD60 in HB cells and confirmed that SNORD60 guides the Nm installation at the A4352 site of KCP mRNA. Moreover, KCP was found to competitively bind SNORD60, thereby preventing its modification of the canonical 28S rRNA target.

Investigating the impact of RNA modifications on mRNA fate is crucial for understanding post-transcriptional regulation in disease and for developing novel therapeutics [29]. Various RNA modifications and sequence-encoded features have been implicated in modulating RNA metabolism; however, the crosstalk between these two layers of regulation remains poorly understood. For example, a previous study demonstrated that m^7^G modification of miRNA precursors counteracts G-quadruplex structures that otherwise inhibit their processing and maturation [30]. Recent work has also shown that internal Nm modifications on mRNAs can influence mRNA decay and expression levels by shortening the 3′-UTR or by stabilizing alternative conformational states [31, 32]. In this study, we addressed how an Nm site located within the coding sequence (CDS) of a specific mRNA negatively regulates its stability. RNA G-quadruplexes (rG4s)—four-stranded secondary structures formed in guanine-rich sequences—perform multifaceted physiological functions, including the regulation of translation [21], mRNA alternative splicing [33], gene silencing [34], and mRNA decay [35]. We identified a thermostable rG4 structure within the CDS of KCP mRNA and confirmed both its formation and its positive role in mRNA stabilization. Furthermore, we demonstrated that SNORD60-guided Nm on KCP mRNA recruits the rG4-specific helicase DDX5, which resolves this structure and promotes KCP mRNA decay in an rG4-dependent manner.

Ferroptosis is a form of regulated cell death driven by iron-mediated oxidative damage and has been implicated in both the pathogenesis and therapeutic response of various cancers [36]. Several experimental compounds—such as erastin and RSL3—and approved drugs, including sorafenib, sulfasalazine, and statins, can induce ferroptotic death in certain cancer cells [18, 37]. Here, we show that SNORD60 plays a key role in enhancing the sensitivity of HB cells to erastin and RSL3 by regulating SLC7A11. ATF4 is a well-characterized transcription factor that activates SLC7A11, a known negative regulator of ferroptosis [23, 38]. In HB, abnormal upregulation of SLC7A11 has been reported to confer resistance to ferroptosis [10]. We found that SNORD60-mediated depletion of KCP significantly inhibited ATF4-driven transcription of SLC7A11 in HB cells. Although the role of KCP in ATF4/SLC7A11 signaling has not previously been reported, numerous studies have shown that the secreted KCP protein enhances bone morphogenetic protein (BMP) signaling—an upstream activator of ATF4 [39, 40]—by promoting ligand–receptor interactions [41, 42]. Here, our findings propose a novel regulatory mechanism in which dysregulation of the SNORD60/KCP axis activates the ATF4/SLC7A11 signaling pathway, thereby inhibiting ferroptosis and promoting HB progression. Thus, combining SNORD60/KCP targeting and ferroptosis induction may be a potential HB treatment strategy. Further studies will be necessary to elucidate the molecular mechanism underlying this regulation and the efficacy of such combined therapy.

## Conclusions

In summary, this study reveals a non-canonical role of SNORD60 in HB pathogenesis, regulating ferroptosis through the KCP–ATF4–SLC7A11 axis. We identified a thermostable rG4 structure within KCP mRNA that stabilizes the transcript. SNORD60 installs Nm at the A4352 site of KCP mRNA and recruits the rG4-specific helicase DDX5 to unwind this structure, thereby promoting KCP mRNA decay. Consequently, aberrant upregulation of KCP in HB cells activates ATF4-mediated transcription of SLC7A11, enhancing ferroptosis resistance and driving malignant progression. Functionally, this SNORD60-mediated pathway sensitizes HB cells to ferroptosis inducers such as erastin and RSL3. Given the growing recognition of ferroptosis induction as a promising cancer therapy, targeting the SNORD60/KCP/SLC7A11 axis to enhance ferroptosis sensitivity may offer a novel therapeutic strategy for HB patients. Furthermore, plasma KCP is a promising diagnostic and prognostic biomarker for HB.

## Materials and Methods

### HB clinical specimens

40 pairs of HB tissues and adjacent non-tumor liver tissues were collected from patients who underwent hepatectomy without receiving preoperative chemotherapy at Shanghai Children’s Medical Center. Non-tumor liver tissue samples, resected approximately 3 cm from the tumor margin, were confirmed to be free of tumor cells by two independent pathologists. Comprehensive clinicopathological data were available for all patients. The study was approved by the Ethics Committee of Shanghai Children’s Medical Center, and written informed consent was obtained from each participant.

### Cell culture and treatment

Two human HB cell lines, HepG2 and HuH6, were obtained from the Type Culture Collection of the Chinese Academy of Sciences. HepG2 cells were cultured in MEM (HyClone, SH30265.01), while HuH6 cells were maintained in DMEM (Gibco Laboratories, C11995500BT). Both media were supplemented with 10% fetal bovine serum (FBS) and 1% penicillin–streptomycin, and cells were incubated at 37 °C in a 5% CO_2_ incubator. For RNA decay assays, cells were treated with 5 μg/mL actinomycin D (MedChemExpress, HY-17559) and harvested at 0, 30, 60, and 120 min. For ferroptosis-related assays, cells were treated with DMSO, erastin, or RSL3 for the indicated durations.

### RNA extraction and qRT‒PCR

Total RNA was extracted from liver tissues or cell lysates using TRIzol reagent (Invitrogen, 15596026). cDNA was synthesized from 1 μg of total RNA using the PrimeScript™ RT reagent kit with gDNA Eraser (TaKaRa Bio, RR047A). Quantitative PCR was performed using Taq Pro Universal SYBR qPCR Master Mix (Vazyme, Q712) to measure the expression levels of SNORD60 and target mRNAs. Relative expression was calculated using the 2^−ΔΔCT^ method. The ΔΔCT method was adopted for Spearman rank correlation analysis [43]. SNORD60 expression was normalized to U6 snRNA, while other mRNA levels were normalized to 18S rRNA. Primer sequences used in this study are listed in Table S1.

### Detection of 2’-O-methylation in KCP mRNA by RTL-P

To detect the 2’-O-methylated site in KCP mRNA, reverse transcription was performed in a 25 μL reaction mixture containing 100 ng of total RNA, either 4 μM or 1 mM dNTPs, and specific RT primers for KCP. The mixture was denatured at 70 °C for 5 min and immediately placed on ice. After annealing at 42 °C for 10 min, 0.5 U of RNasin ribonuclease inhibitor (Promega, N2111) and 200 U of M-MLV reverse transcriptase (Takara, 2641) were added. The reaction was incubated at 42 °C for 1 h, followed by heat inactivation at 75 °C for 15 min. The cDNA was amplified using PrimeSTAR® Max DNA Polymerase (Takara, R045) with specific primers for KCP. PCR products were separated on a 2% agarose gel and visualized using a gel imaging system (Tanon).

### Circular dichroism (CD) spectroscopy

All RNA oligomers (10 μM) were dissolved in 10 mM Tris–HCl buffer (pH 7.5) containing either 150 mM KCl or LiCl, and then refolded through a heat–cooling process prior to measurement. CD spectra were recorded from 225 to 325 nm at 25 °C using a JASCO J-1500 CD spectrophotometer with a 10 mm path-length cuvette. Data were normalized to molar residue ellipticity and analyzed using Spectra Manager Suite (JASCO Software). For thermal melting experiments, CD signals at 262 nm were monitored while gradually increasing the temperature from 23 °C to 95 °C at a rate of 2 °C/min. The melting temperature (Tm) was defined as the temperature at which half of the maximum signal increase was observed.

### Tumor xenograft experiment

Four-week-old male nude mice were purchased from Shanghai Super-B&K Laboratory Animal Corporation (Shanghai, China). A total of 1 × 10⁷ HuH6 cells stably expressing LV-NC or LV-SNORD60 were orthotopically injected into the liver of each mouse. After 28 days, the mice were sacrificed, and tumors were harvested, weighed, and photographed. Ki67 staining of tumor tissues was performed by Runnerbio (Shanghai, China). All animal experiments were conducted in accordance with the Guidelines for the Care and Use of Animals for Scientific Research.

### Nm-Seq

Nm-seq was performed on poly(A)-tailed RNA extracted from SNORD60-overexpressing HepG2 cells following a previously described protocol [44]. Briefly, 10 μg of poly(A)-selected RNA was fragmented using RNA Fragmentation Reagents (Thermo Fisher Scientific, AM8740) at 95 °C for 5 min. Fragmented RNA underwent 3′ end repair with Antarctic Phosphatase (New England Biolabs, M0289) at 37 °C for 30 min. Next, the samples underwent eight cycles of oxidation–elimination–dephosphorylation (OED), followed by 5′ phosphorylation. After a final oxidation–elimination step, RNA was subjected to library construction using the NEBNext® Small RNA Library Prep Set for Illumina (New England Biolabs, E7300). Sequencing was carried out on the Illumina HiSeq-PE150 platform. Raw sequencing reads were trimmed to remove standard adapters (“AGATCGGAAGAGCACACGTCT”) and low-quality bases using Cutadapt. Cleaned reads were aligned to the human genome (hg38) and transcriptome using TopHat. Positive Nm sites were identified based on the following criteria:

1 $${\mathrm{Chi}}({{\rm{D}}}_{\mathrm{base}}\_{\mathrm{Nm}}{,{\rm{D}}}_{\mathrm{Ave}}\_{\mathrm{Nm}}{,{\rm{D}}}_{\mathrm{base}}\_{\mathrm{input}}{,{\rm{D}}}_{\mathrm{Ave}}\_{\mathrm{input}}):{{\log2}}({\mathrm{ratio}})\ge {{1}}\,\& \,{\mathrm{Depth}}\,{\mathrm{of}}\,{\mathrm{the}}\,{\mathrm{base}}\ge 5.$$

2 $${\mathrm{Chi}}({{\rm{D}}}_{\mathrm{base}}\_{\mathrm{Nm}}{,{\rm{D}}}_{\mathrm{Sum}}\_{\mathrm{Nm}}{,{\rm{D}}}_{\mathrm{Ave}}\_{\mathrm{Nm}}{,{\rm{D}}}_{\mathrm{Sum}}\_{\mathrm{Nm}}):{\rm{p}}-{\mathrm{value}} < 0.01\,\& \,{{\log2}}({{\rm{D}}}_{\mathrm{base}}\_{\mathrm{Nm}}{/{\rm{D}}}_{\mathrm{Ave}}\_{\mathrm{Nm}})\ge{2}.$$

### Statistical analysis

All data were analyzed using SPSS Statistics (IBM Corporation) and GraphPad Prism (GraphPad Software). Results from experiments with a minimum of three biological replicates are presented as mean ± SD. For comparisons between two groups, either an independent or paired-samples t-test was used, as appropriate. For comparisons among more than two groups, one-way ANOVA (Bonferroni’s test or Dunnett’s test) was performed. Receiver Operating Characteristic (ROC) curves were generated to assess the diagnostic value of KCP. Fisher’s exact test was used to evaluate the association between KCP expression and clinicopathological characteristics of HB patients. Statistical significance is shown as *p < 0.05, **p < 0.01, ***p < 0.001.

For further detailed materials and methods, see the supplementary materials and methods.

## Supplementary information

Supplementary figure legends

Supplementary materials and methods

Figure S1

Figure S2

Figure S3

Figure S4

Table S1

Table S2

Table S3

Table S4

Table S5

Table S6

Original Western blotting images

## Data Availability

The data generated in this study are available within the article and its supplementary data files.
