# Supplementary material for: SNORD60-mediated 2′-O-methylation of KCP enhances ferroptosis sensitivity in hepatoblastoma
Source: Cell Death Discov. 2026 May 22;12:304. doi: 10.1038/s41420-026-03160-5 (PMC13369958; doi:10.1038/s41420-026-03160-5)
Supplement: Supplementary file 1 — Supplementary figure legends [file 41420_2026_3160_MOESM1_ESM.docx]

**Supplementary figure legends**

**Figure S1:** **SNORD60 overexpression induces ferroptotic cell death.** (A) Heatmap clustering of snoRNA sequencing results for 4 pairs of HB and NT tissues. (B) Apoptosis in HepG2/LV-NC and HepG2/LV-SNORD60 cells were assessed by flow cytometry. (C) Apoptosis in HuH6/LV-NC and HuH6/LV-SNORD60 cells were assessed by flow cytometry. (D) Volcano plot showing significantly differentially expressed genes in HepG2/LV-SNORD60 cells compared with HepG2/LV-NC cells. (E) KEGG pathway enrichment analysis of significantly differentially expressed genes. (F) The relative Fe^3+^/Fe^2+^ ratio in HB cells overexpressing SNORD60. (G) The relative GSH/GSSG ratio in HB cells overexpressing SNORD60.

**Figure S2: SNORD60-mediated downregulation of KCP is rescued by DDX5 knockdown.** (A-B) Relative mRNA levels of DDX5 in HB cells transfected with siRNAs targeting DDX5 (A) or with OE-NC or OE-DDX5 plasmids (B) were measured by qRT‒PCR. (C) Relative mRNA level of DDX5 in HB cells was measured by qRT‒PCR after treatment with DC3 or RR82. (D) Relative mRNA level of KCP in HB cells co-transfected with siRNAs targeting DDX5 and SNORD60-overexpressing plasmids was measured by qRT‒PCR. (E-G) Detection of cell proliferative activity by CCK-8 assay (E-F) and colony formation assay (G). Cells were subjected to the same treatment as in (D).

**Figure S3: SNORD60 promotes HB cell proliferation by downregulating KCP expression.** (A) Relative mRNA level of KCP in HB cells co-transfected with SNORD60-overexpressing plasmids and KCP-overexpressing plasmids was measured by qRT‒PCR. (B-D) Detection of cell proliferative activity by CCK-8 assay (B-C) and colony formation assay (D). Cells were subjected to the same treatment as in (A).

**Figure S4: SNORD60/KCP axis promotes ferroptosis by suppressing the ATF4/SLC7A11 signaling pathway.** (A-C) Western blotting analyses of the ferroptosis-associated proteins GPX4 and ACSL4 in HB cells overexpressing SNORD60 (A), cells with KCP knockdown (B), and cells with ATF4 knockdown (C). Relative densitometry was performed with ImageJ.
