# Supplementary material for: SNORD60-mediated 2′-O-methylation of KCP enhances ferroptosis sensitivity in hepatoblastoma
Source: Cell Death Discov. 2026 May 22;12:304. doi: 10.1038/s41420-026-03160-5 (PMC13369958; doi:10.1038/s41420-026-03160-5)
Supplement: Supplementary file 2 — Supplementary materials and methods [file 41420_2026_3160_MOESM2_ESM.docx]

### **Supplementary materials and methods**

Cell proliferation assay

To assess cell proliferation, HB cells were seeded into 96-well plates at a density of 1,000 cells per well. At the indicated time points, the culture medium was removed, and 100 μL of fresh medium containing 10 μL of CCK-8 reagent (Beyotime Biotechnology, C0043) was added to each well. After a 2-hour incubation at 37 °C, the absorbance at 450 nm was measured using a Synergy2 multimode microplate reader (BioTek Instruments). For colony formation assays, 1,000 cells were plated in 12-well plates and cultured for 7–10 days. The cells were then fixed with 4% paraformaldehyde and stained with 0.1% crystal violet. Colonies were photographed and quantified using ImageJ software.

Flow cytometry

Lipid peroxidation and cell death were assessed according to the manufacturer’s instructions. For lipid peroxidation detection, cells were harvested and stained with BODIPY 581/591 C11 (MedChemExpress, HY-D1301) for 30 min at room temperature in the dark, then washed twice with PBS. The stained cells were analyzed by flow cytometry (BD Biosciences). For cell death analysis, cells were incubated with propidium iodide (Invitrogen, P3566) for 15 min at room temperature in the dark and analyzed by flow cytometry. For apoptosis analysis of HB/LV-NC and HB/LV-SNORD60 cells, an Annexin V–APC/7-AAD apoptosis kit (MultiSciences Biotech, AP105) was used. Cells were harvested, stained with 5 μL Annexin V–APC and 10 μL 7-AAD for 5 min at room temperature in the dark, and analyzed by flow cytometry.

Transfection of siRNA or plasmid

siRNAs targeting KCP, DDX5, or ATF4 were purchased from GenePharma (Shanghai, China). siRNA sequences used in this study are listed in Table S1. Expression vectors for SNORD60, KCP, and DDX5 were obtained from Ke Lei Biotechnology (Shanghai, China), based on the pcDNA3.1(-) backbone.

To construct pGMLV-SNORD60, the SNORD60 coding sequence was cloned into the pGMLV-6395 lentiviral expression vector (Genomeditech, Shanghai, China). Transfections of siRNA or plasmid were performed using Lipofectamine™ 2000 (Invitrogen, 11668019) according to the manufacturer’s instructions.

RNA immunoprecipitation qPCR (RIP- qPCR)

The RIP assay was performed using the Magna RIP kit (Sigma-Aldrich, 17-700) following the manufacturer’s protocol. Briefly, HB cell pellets were lysed in RIP lysis buffer on ice for 5 min, then incubated overnight at 4 °C with mouse/rabbit IgG, anti-Fibrillarin antibody (Santa Cruz Biotechnology, 374022), or anti-DDX5 antibody (Cell Signaling Technology, 9877) conjugated to protein A/G magnetic beads to enrich RNA-protein complexes. After digestion with Proteinase K, RNA was purified by phenol-chloroform-isoamyl alcohol extraction and ethanol precipitation, then reverse-transcribed into cDNA. Quantitative PCR was performed to determine the %Input of selected RNA candidates using primers listed in Table S1.

RNA pull-down assay

RNA pull-down assays were performed using the Pierce Magnetic RNA-Protein Pull-Down Kit (Thermo Fisher Scientific) according to the manufacturer’s instructions. Biotinylated KCP-WT, KCP-Nm, and reverse probes were obtained from Biosune (Shanghai, China), with the reverse probe serving as a negative control. Briefly, 50 pmol of each biotinylated probe was incubated with streptavidin-coated magnetic beads for 30 min at room temperature. The RNA-bound beads were then incubated with cell lysates overnight at 4 °C. Proteins captured on the beads were analyzed by label-free quantitative proteomics using an Easy nLC 1000 UHPLC system coupled to a Q Exactive HF-X mass spectrometer (Thermo Fisher Scientific). Probe sequences used in this study are listed in Table S1.

Western blotting

Cells were lysed in RIPA lysis buffer (Beyotime Biotechnology, P0013B) on ice for 10 min. Protein samples were separated on 10% SDS-PAGE gels and transferred onto nitrocellulose membranes (Beyotime Biotechnology) using a wet transfer method. Membranes were blocked with QuickBlock™ Protein-Free Blocking Buffer (Beyotime Biotechnology, P0240) for 20 min at room temperature. After blocking, membranes were incubated overnight at 4 °C with primary antibodies, followed by incubation with IRDye anti-mouse/rabbit IgG secondary antibody (LI-COR Biosciences, 926-32210/926-32211) for 30 min at 37 °C. Protein bands were visualized using the Odyssey infrared imaging system (LI-COR Biosciences). The primary antibodies used in this study included: primary anti-β-actin antibody (Abcam, 8226), anti-xCT/SLC7A11 antibody (Cell Signaling Technology, 12691), anti-ATF-4 antibody (Cell Signaling Technology, 11815), anti-GPX4 antibody (Proteintech, 67763-1), anti-ACSL4 antibody (Cell Signaling Technology, 38493), anti-puromycin antibody (Sigma-Aldrich, MABE343).

Immunohistochemistry (IHC) and in situ hybridization (ISH)

IHC was performed on paraffin-embedded tissues or HB tissue microarrays by Runnerbio Corporation (Shanghai, China) following a previously published protocol [1]. Ki67 expression was detected using a primary anti-Ki67 antibody (Abcam, 15580), and KCP expression was detected using a primary anti-KCP antibody (Affinity Biosciences, DF14328). After staining, tissue sections were scanned using a KF-PRO-120 digital pathology slide scanner (Konfoong Bioinformation Tech) and analyzed with K-Viewer digital slide reading software (Konfoong Bioinformation Tech).

For the ISH assay, a digoxin-labeled probe specific to SNORD60 was purchased from BioSune Biotech Corp (Shanghai, China). ISH was performed on HB tissue microarrays, and panoramic scanning was conducted by Shanghai ZuoCheng Bio (Shanghai, China). Staining intensity was evaluated at 200× and 400× magnifications by two specialized pathologists who were blinded to the clinicopathological information.

Enzyme-linked immunosorbent assay (ELISA)

Blood samples from HB patients and healthy controls were collected and centrifuged at 1,000 × *g* for 15 min at 4 °C to separate the plasma. Liver tissues were homogenized in cold PBS and centrifuged at 5,000 × *g* for 10 min to collect the supernatant. The levels of KCP in liver tissues, plasma, and cell culture media were measured using the Kielin Cysteine Rich BMP Regulator ELISA kit (Shanghai Jianglai Biotechnology Co., Ltd., JL23826), following the manufacturer’s instructions.

Measurement of MDA Content

The malondialdehyde (MDA) content in HB cells under the indicated treatments was measured using a lipid peroxidation MDA assay kit (Beyotime, S0131), which is based on the reaction between MDA and thiobarbituric acid. Absorbance was recorded at 532 nm, and MDA levels were calculated from the standard curve generated using the reference standards provided in the kit under identical conditions.

Measurement of iron concentration and the GSH/GSSG ratio

Total iron, Fe²⁺, and Fe³⁺ levels were measured using an iron assay kit (Abcam, ab83366) according to the manufacturer’s instructions. Iron concentration was calculated using the following formula: iron concentration (µM) = iron content (nmol) / sample volume (µL) × dilution factor.

The GSH/GSSG ratio was determined using commercial assay kits for reduced glutathione (GSH) and oxidized glutathione (GSSG) (Solarbio, BC1175 and BC1180, respectively), following the manufacturer’s protocols.

Transcriptomics

mRNA sequencing of SNORD60-overexpressing HepG2 cells was performed by OE Biotech Co., Ltd. (Shanghai, China) using the Illumina NovaSeq 6000 platform with 150 bp paired-end reads, following the manufacturer’s protocols. Raw reads were processed with fastp and low quality reads were removed for clean reads. These clean reads were then aligned to the human reference genome (hg38) using HISAT2. FPKM of each gene was calculated and read counts of each gene were acquired using HTSeq-count. Differential expression analysis was performed using DESeq2. Genes with a fold change > 2 or < 0.5 and a Q-value < 0.05 were considered significantly differentially expressed (DEGs). Gene Ontology (GO) and KEGG pathway enrichment analyses were conducted for DEGs using R software (v3.2.0) to identify significantly enriched biological terms and pathways.

**Reference**

[1] J. Zhu, S. Mao, N. Zhen, G. Zhu, Z. Bian, Y. Xie, X. Tang, M. Ding, H. Wu, J. Ma, Y. Zhu, F. Sun, Q. Pan, SNORA14A inhibits hepatoblastoma cell proliferation by regulating SDHB-mediated succinate metabolism, Cell death discovery, 9 (2023) 36.
