# Supplementary figures and images for: SNORD60-mediated 2′-O-methylation of KCP enhances ferroptosis sensitivity in hepatoblastoma
Source: Cell Death Discov. 2026 May 22;12:304. doi: 10.1038/s41420-026-03160-5 (PMC13369958; doi:10.1038/s41420-026-03160-5)

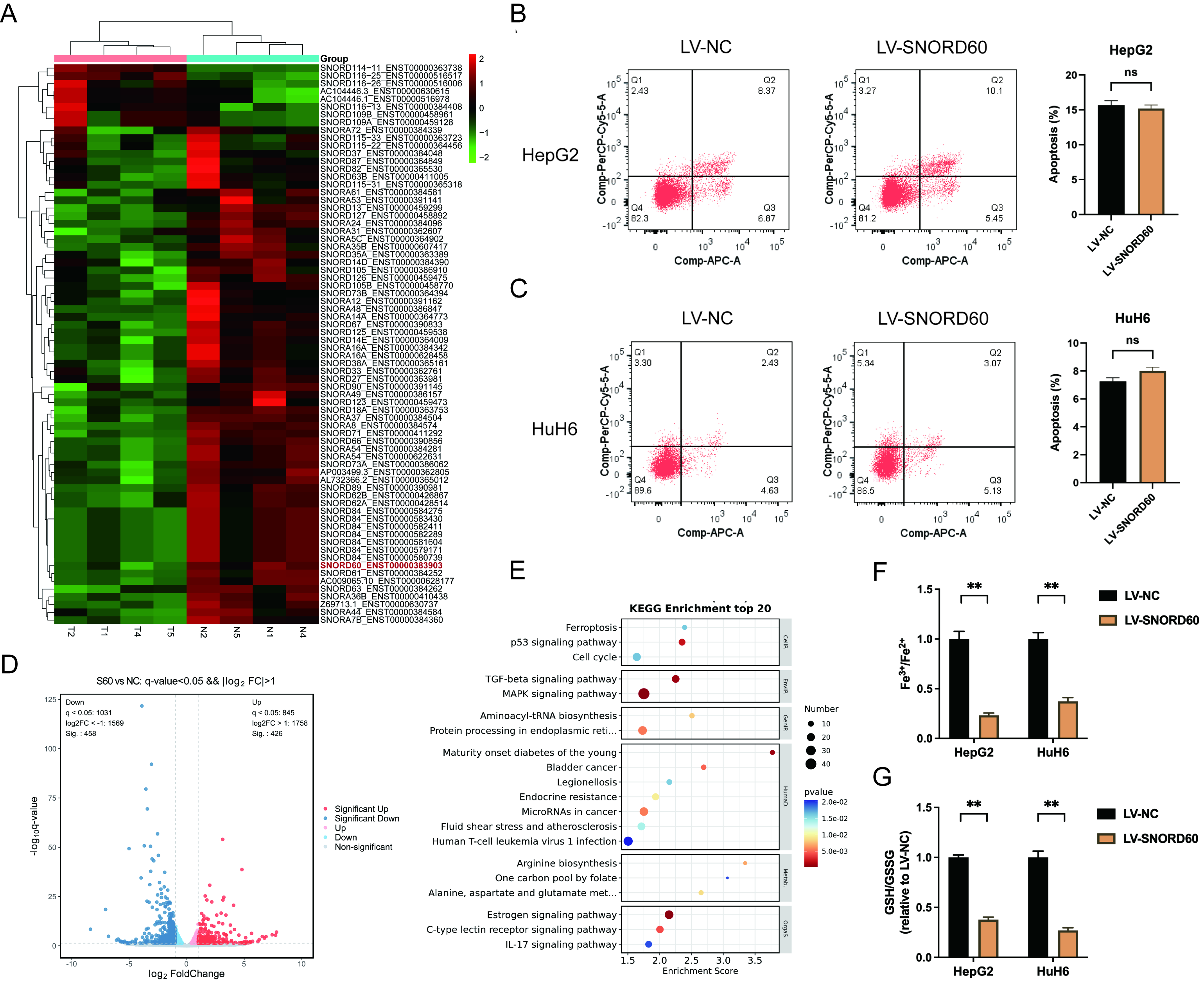

Supplement: Supplementary file 3 — Figure S1 [file 41420_2026_3160_MOESM3_ESM.tif]

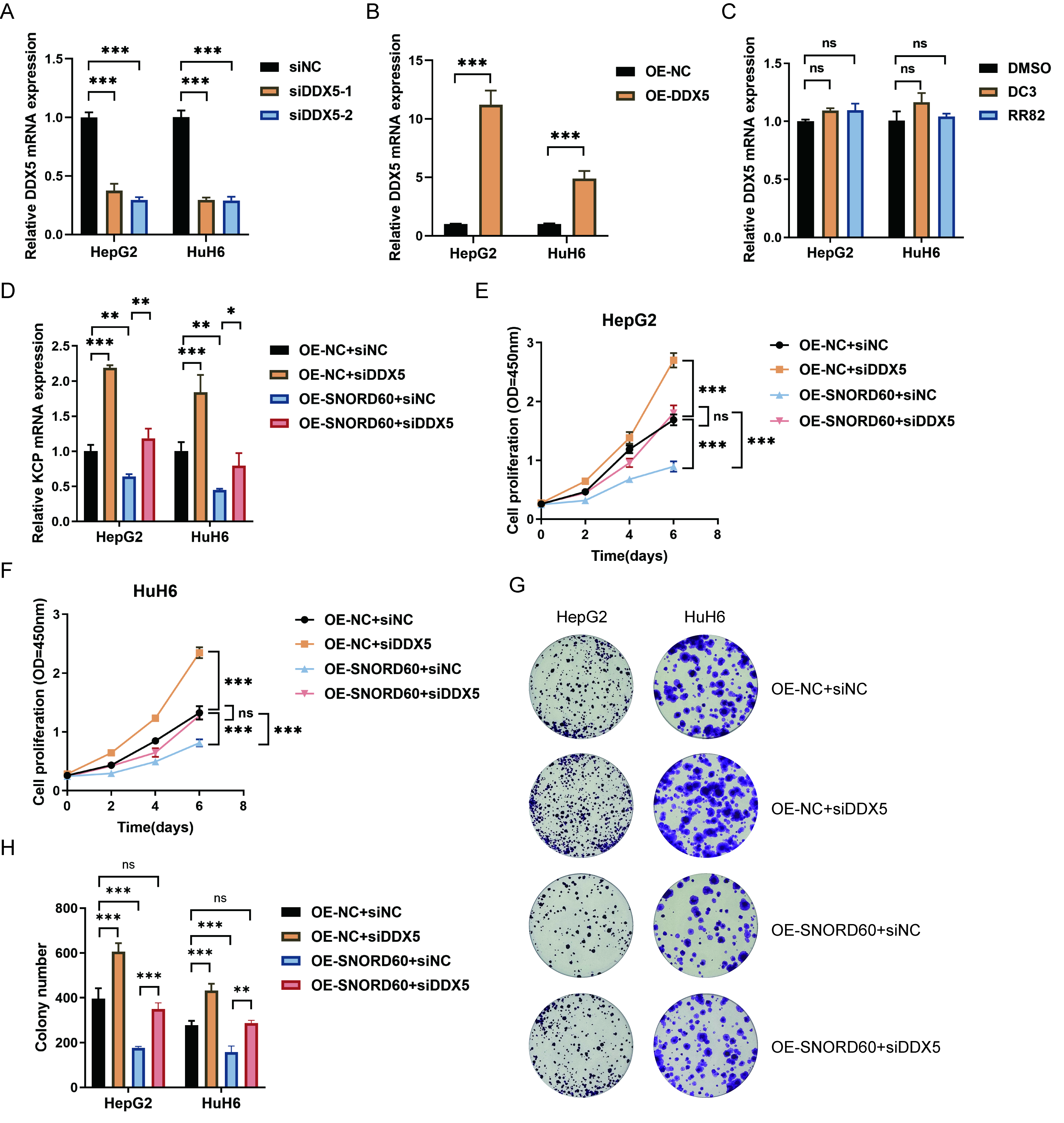

Supplement: Supplementary file 4 — Figure S2 [file 41420_2026_3160_MOESM4_ESM.tif]

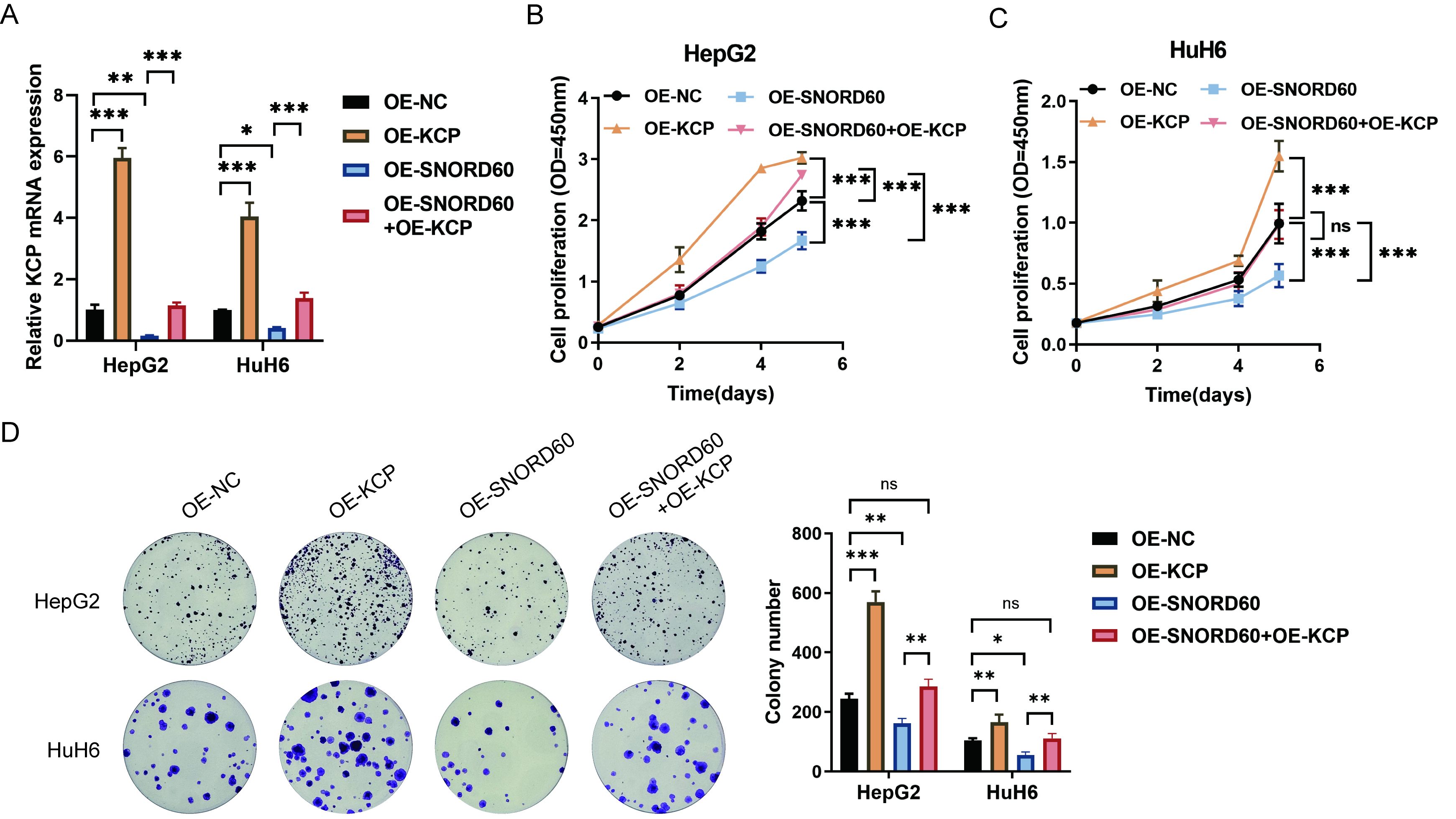

Supplement: Supplementary file 5 — Figure S3 [file 41420_2026_3160_MOESM5_ESM.tif]

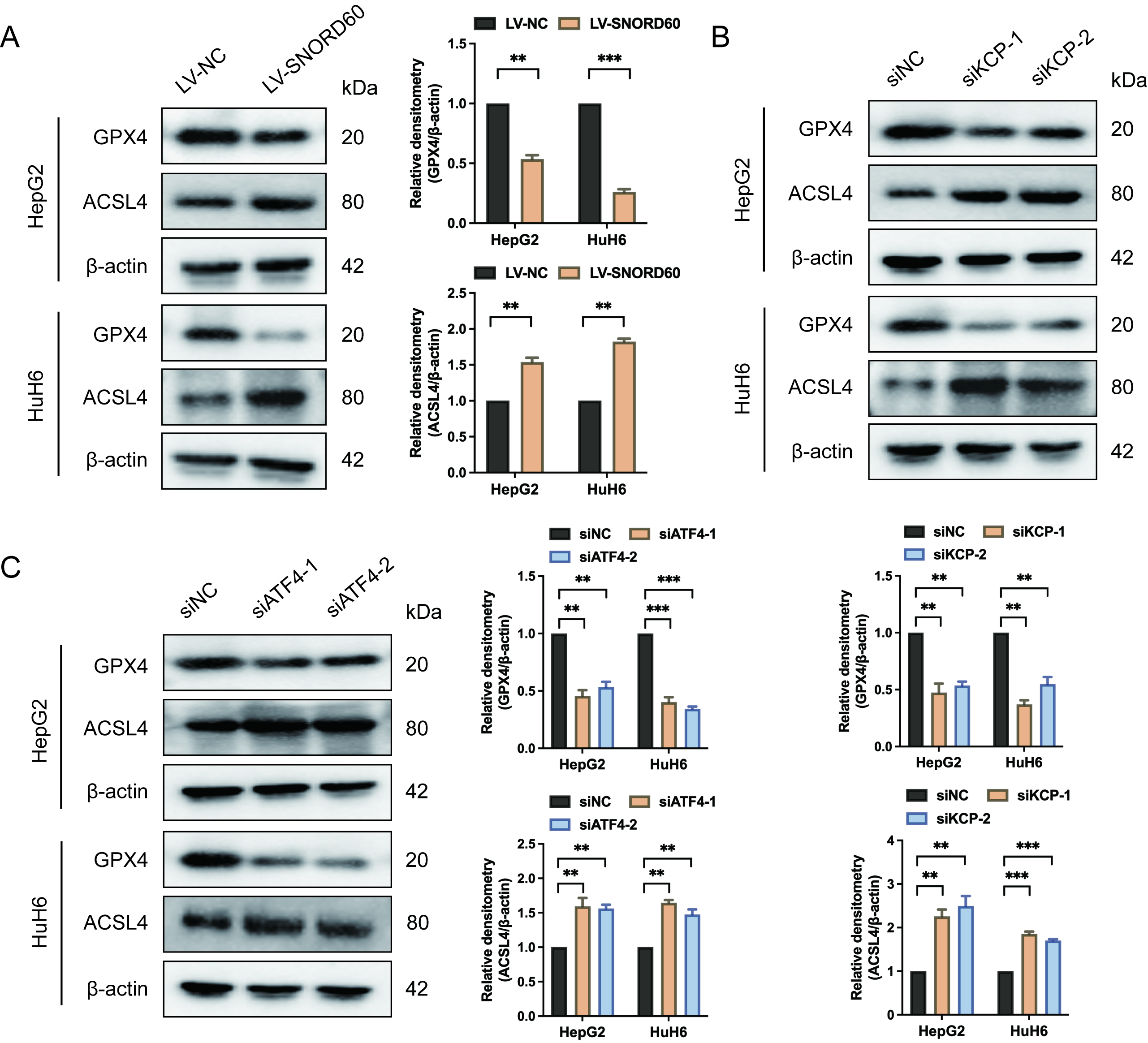

Supplement: Supplementary file 6 — Figure S4 [file 41420_2026_3160_MOESM6_ESM.tif]
