# Supplementary material for: SNORD60-mediated 2′-O-methylation of KCP enhances ferroptosis sensitivity in hepatoblastoma
Source: Cell Death Discov. 2026 May 22;12:304. doi: 10.1038/s41420-026-03160-5 (PMC13369958; doi:10.1038/s41420-026-03160-5)
Supplement: Supplementary file 11 — Table S5 [file 41420_2026_3160_MOESM11_ESM.docx]

**Table S5: The clinical information and pathological characteristics of 40 HB patients.**

| **Variable** | **n** |
| --- | --- |
| **Age at Diagnosis (month)** |  |
| ≥24/<24 | 15/25 |
| **Sex** |  |
| Male/Female | 24/16 |
| **AFP at Diagnosis (ng/ml)** |  |
| ≥1200/<1200 | 33/7 |
| **AFP at Final Detection (ng/ml)** | |
| ≥5/<5/NA | 25/11/4 |
| **Histology** |  |
| MIX/E/NA | 17/8/15 |
| **PRETEXT** |  |
| I-II/III-IV/NA | 14/19/7 |
| **Tumor Size(cm^3^)** |  |
| ≥500/<500 | 23/17 |
| **Metastasis** |  |
| YES/NO | 11/29 |
