# Supplementary material for: SNORD60-mediated 2′-O-methylation of KCP enhances ferroptosis sensitivity in hepatoblastoma
Source: Cell Death Discov. 2026 May 22;12:304. doi: 10.1038/s41420-026-03160-5 (PMC13369958; doi:10.1038/s41420-026-03160-5)
Supplement: Supplementary file 12 — Table S6 [file 41420_2026_3160_MOESM12_ESM.docx]

**Table S6: The correlation analysis of KCP expression level and clinicopathological characteristics of 40 HB patients.**

| **HB (n=40)** | **KCP expression** | | **p-value** |
| --- | --- | --- | --- |
|  | **Low** | **High** |  |
| **Age at Diagnosis** |  |  |  |
| ≥24 month | 7 | 8 | 1.000 |
| <24 month | 13 | 12 |  |
| **Sex** |  |  |  |
| Male | 11 | 13 | 0.748 |
| Female | 9 | 7 |  |
| **AFP at Diagnosis** |  |  |  |
| ≥1200 ng/ml | 16 | 17 | 1.000 |
| <1200 ng/ml | 4 | 3 |  |
| **AFP at Final Detection** |  |  |  |
| ≥5 ng/ml | 11 | 14 | 0.651 |
| <5 ng/ml | 7 | 4 |  |
| NA | 2 | 2 |  |
| **Histology** |  |  |  |
| MIX | 9 | 8 | 0.841 |
| E | 3 | 5 |  |
| NA | 8 | 7 |  |
| **PRETEXT** |  |  |  |
| I-II | 10 | 4 | **0.022** |
| III-IV | 5 | 14 |  |
| NA | 5 | 2 |  |
| **Tumor Size** |  |  |  |
| ≥500 cm^3^ | 7 | 16 | **0.010** |
| <500 cm^3^ | 13 | 4 |  |
| **Metastasis** |  |  |  |
| YES | 2 | 9 | **0.031** |
| NO | 18 | 11 |  |
