# Supplementary material for: SNORD60-mediated 2′-O-methylation of KCP enhances ferroptosis sensitivity in hepatoblastoma
Source: Cell Death Discov. 2026 May 22;12:304. doi: 10.1038/s41420-026-03160-5 (PMC13369958; doi:10.1038/s41420-026-03160-5)
Supplement: Supplementary file 13 — Original Western blotting images [file 41420_2026_3160_MOESM13_ESM.pdf]

Original images

Figure 3C

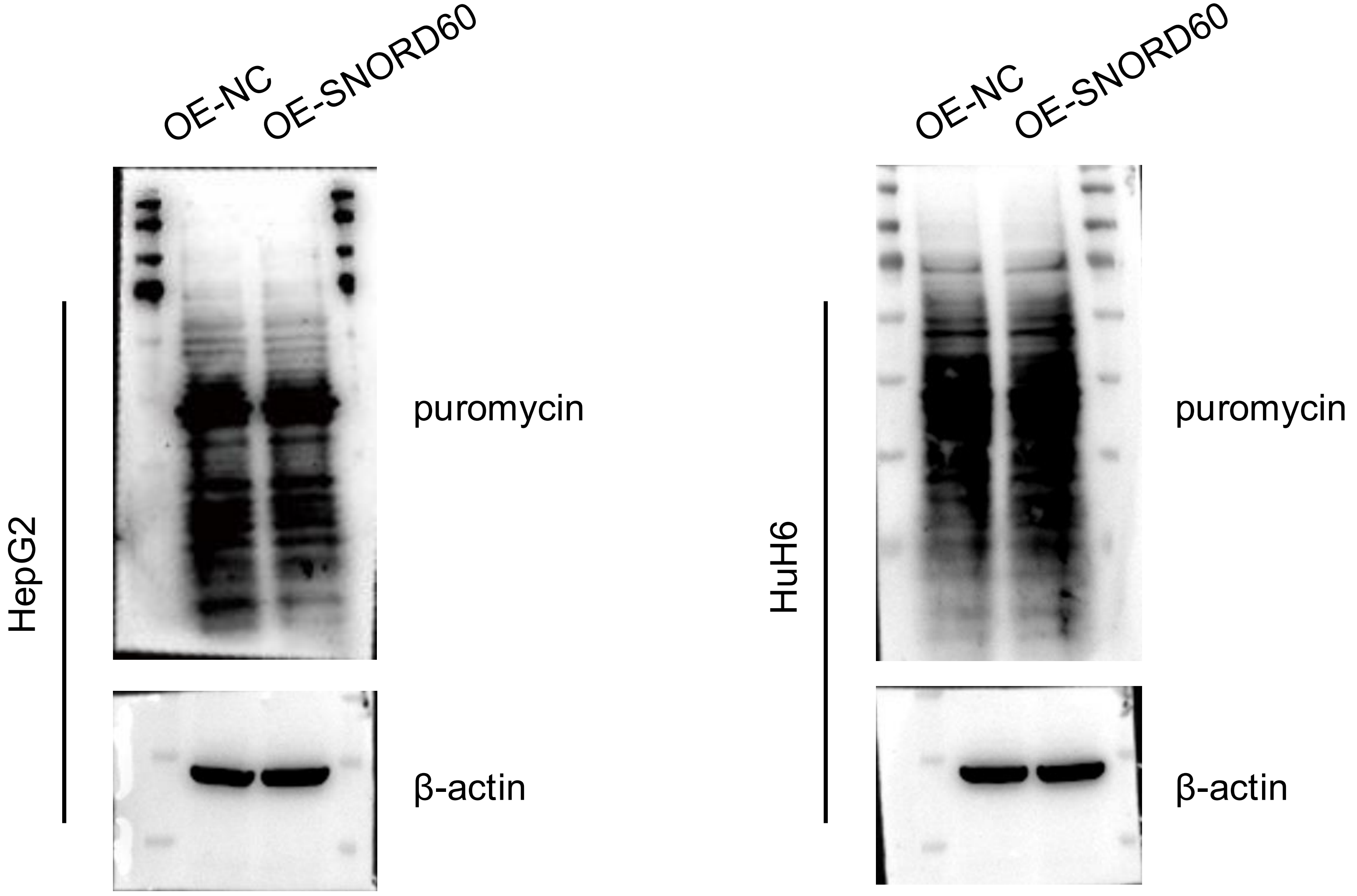

Figure 4F

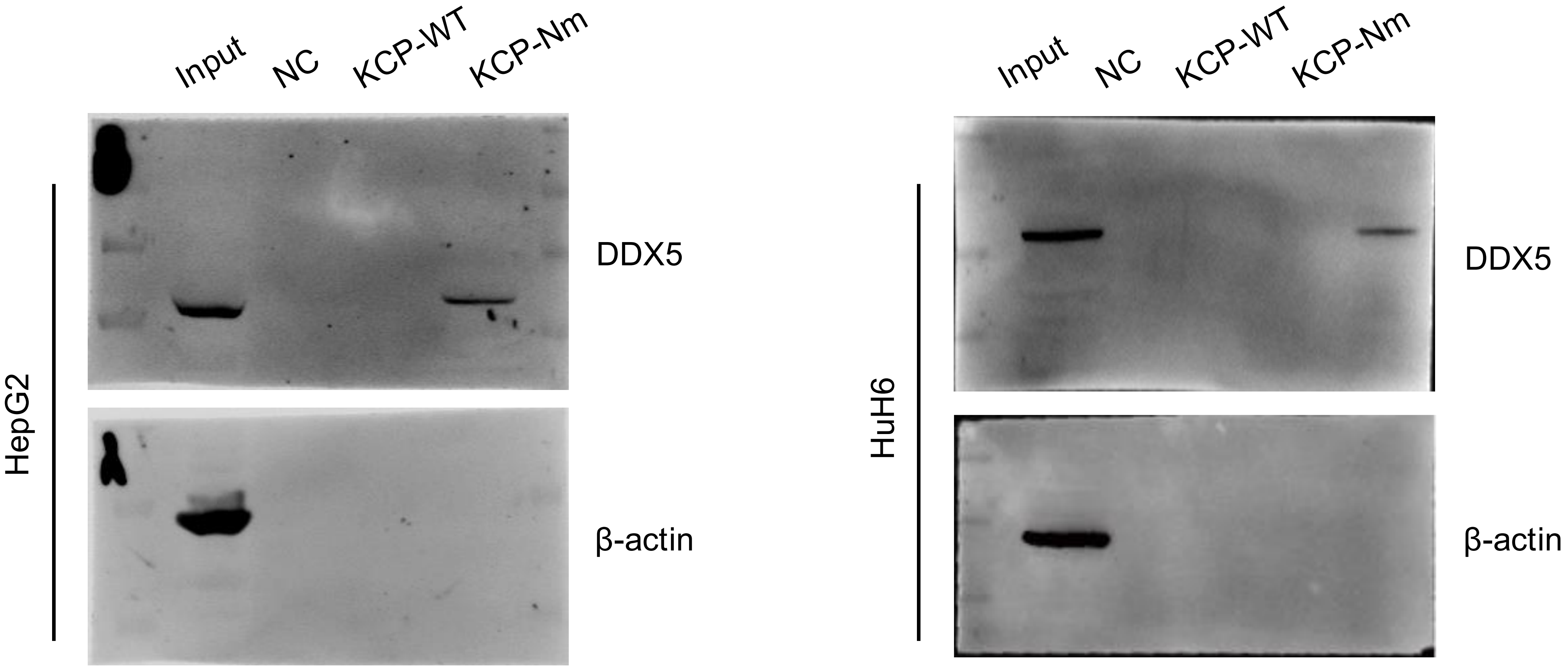
Input
NC
KCP-WT
KCP-Nm
HepG2
DDX5
β-actin
Input
NC
KCP-WT
KCP-Nm
HuH6
DDX5
β-actin

Figure 6C

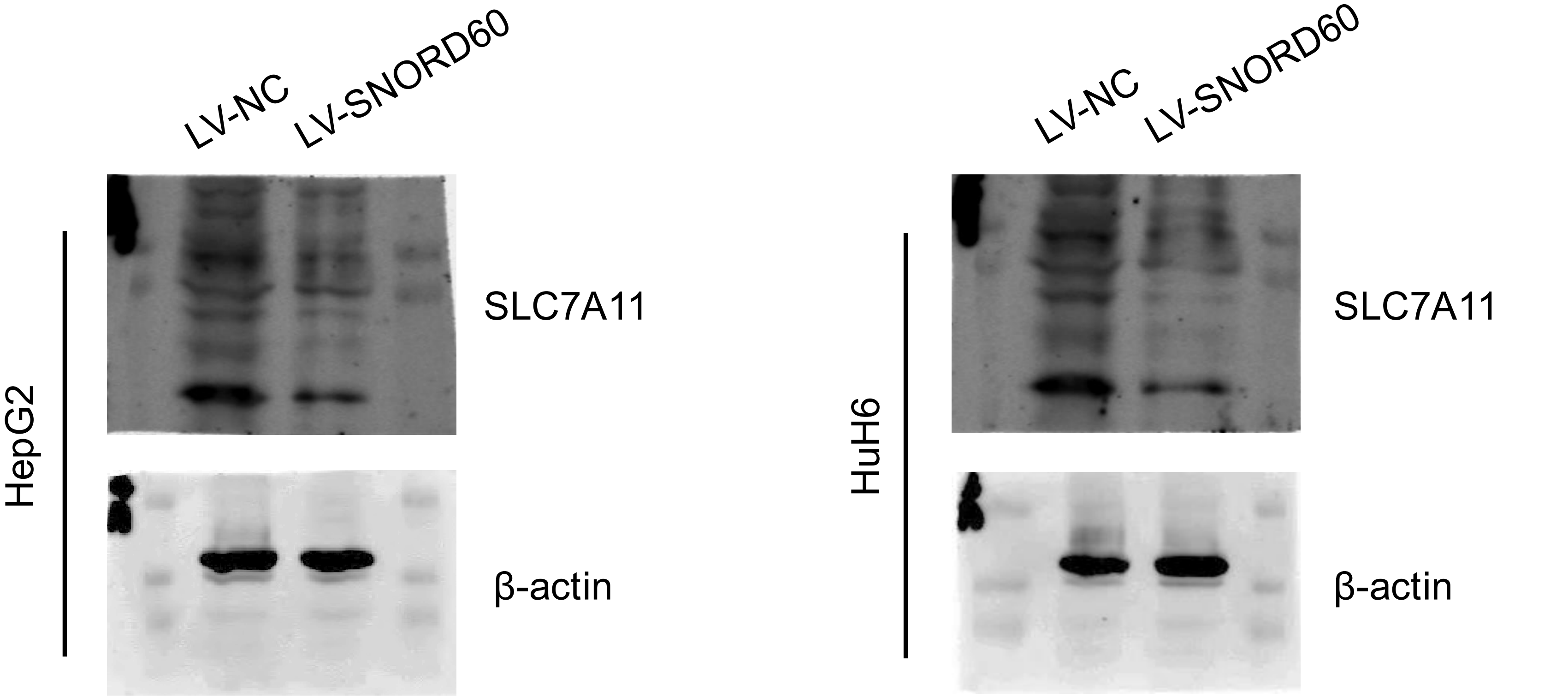
LV-NC
LV-SNORD60
HepG2
SLC7A11
β-actin
LV-NC
LV-SNORD60
HuH6
SLC7A11
β-actin

Figure 6E

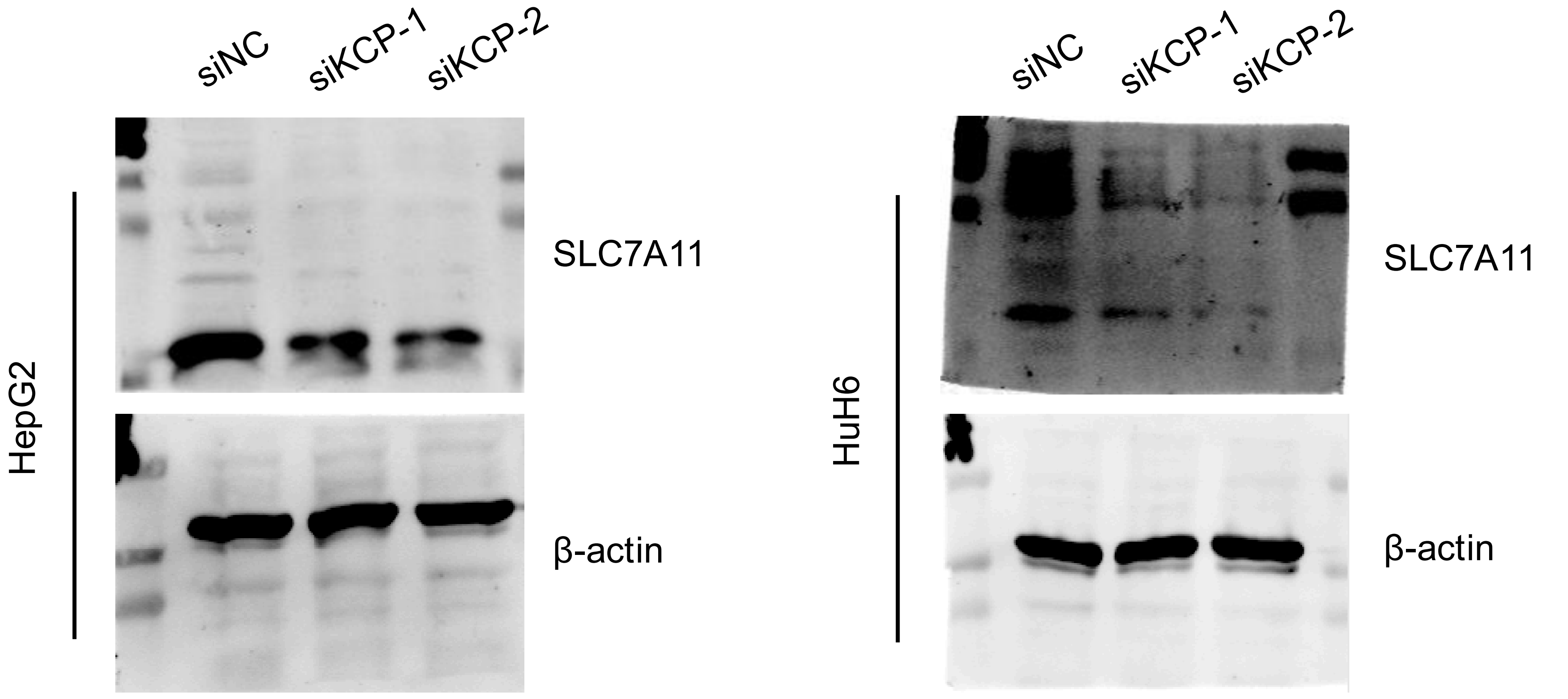
siNC
siKCP-1
siKCP-2
SLC7A11
β-actin
HepG2
siNC
siKCP-1
siKCP-2
SLC7A11
β-actin
HuH6

Figure 6H

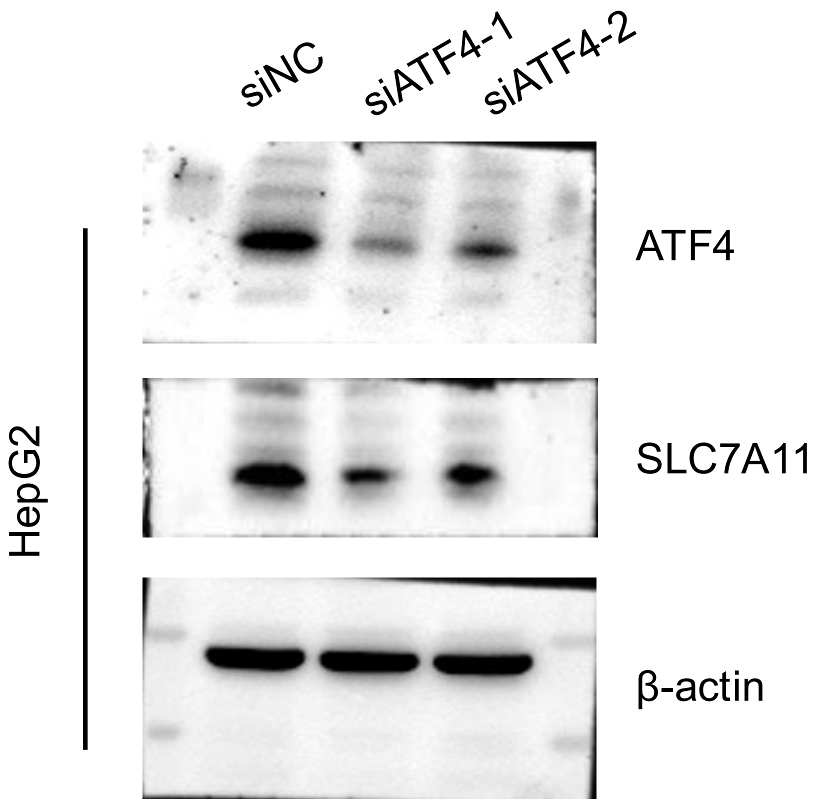

Figure 6I

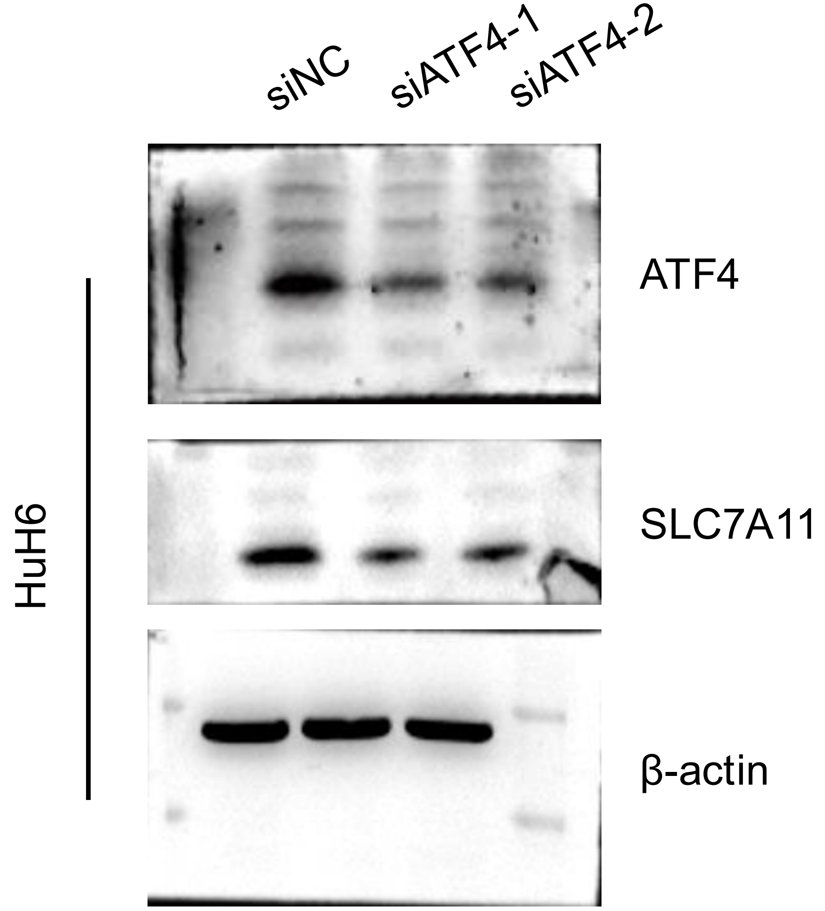

Figure S4A

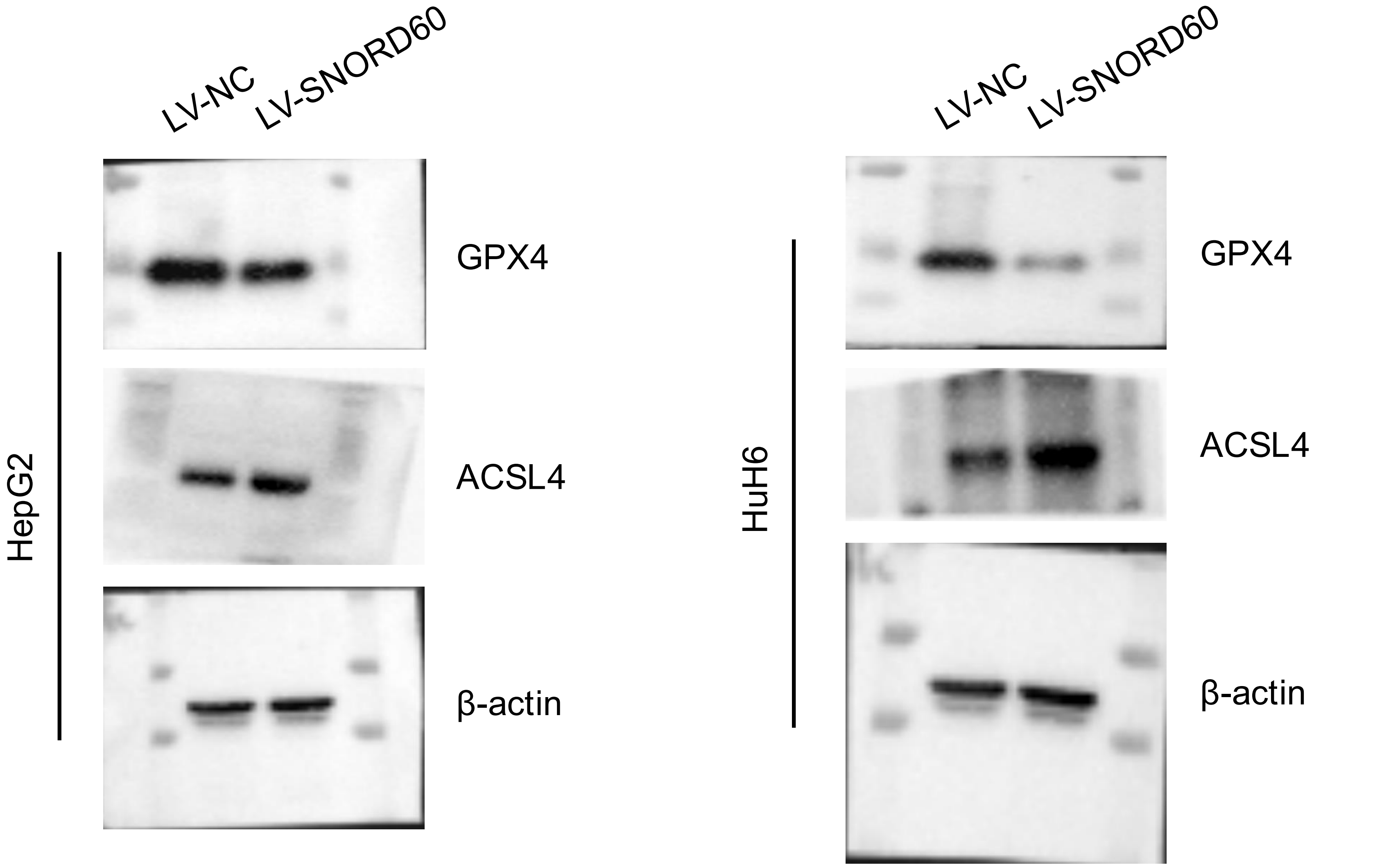
LV-NC
LV-SNORD60
GPX4
ACSL4
β-actin
HepG2
LV-NC
LV-SNORD60
GPX4
ACSL4
β-actin
HuH6

Figure S4B

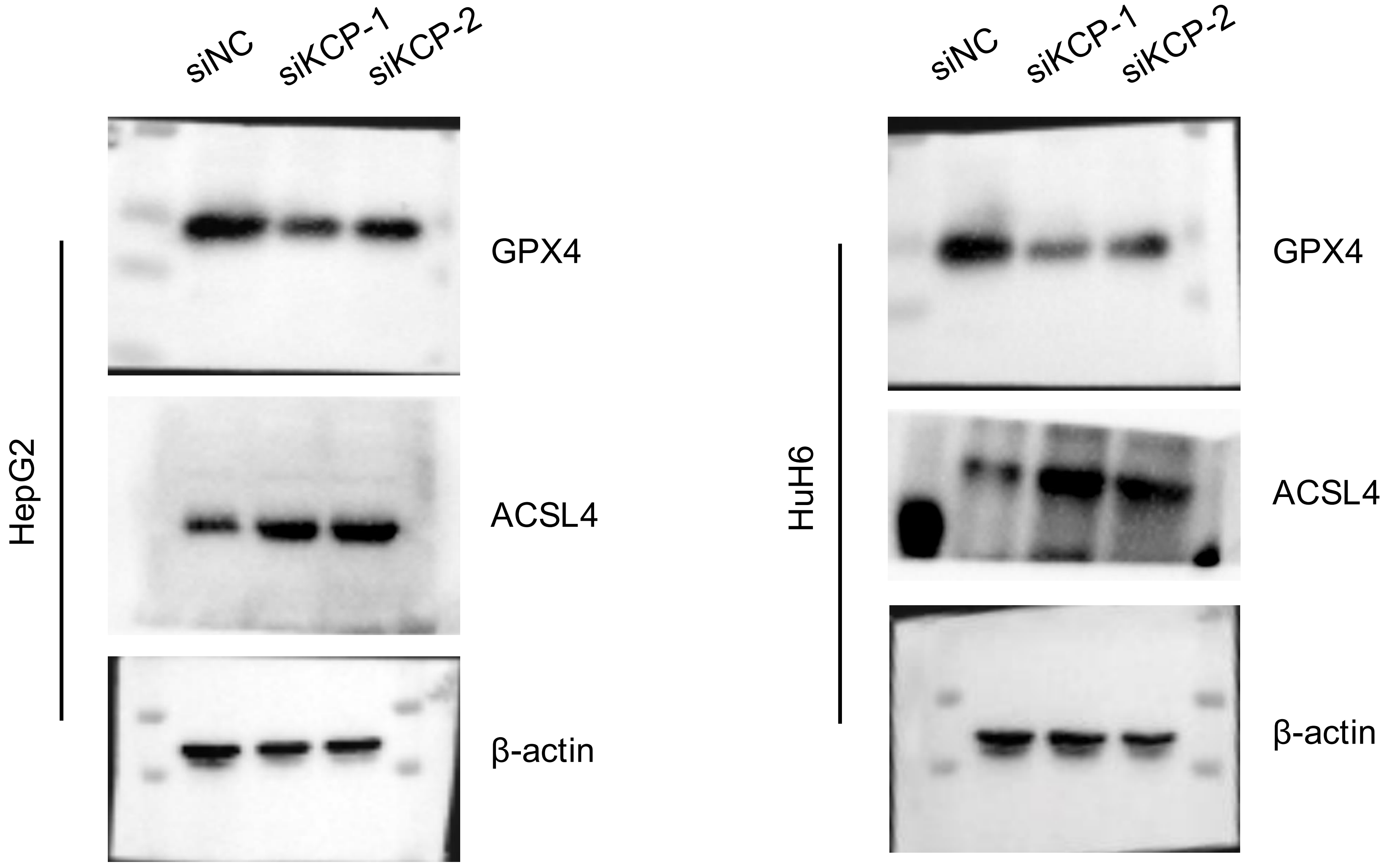

siNC
siKCP-1
siKCP-2
GPX4
ACSL4
β-actin
HepG2
siNC
siKCP-1
siKCP-2
GPX4
ACSL4
β-actin
HuH6

Figure S4C

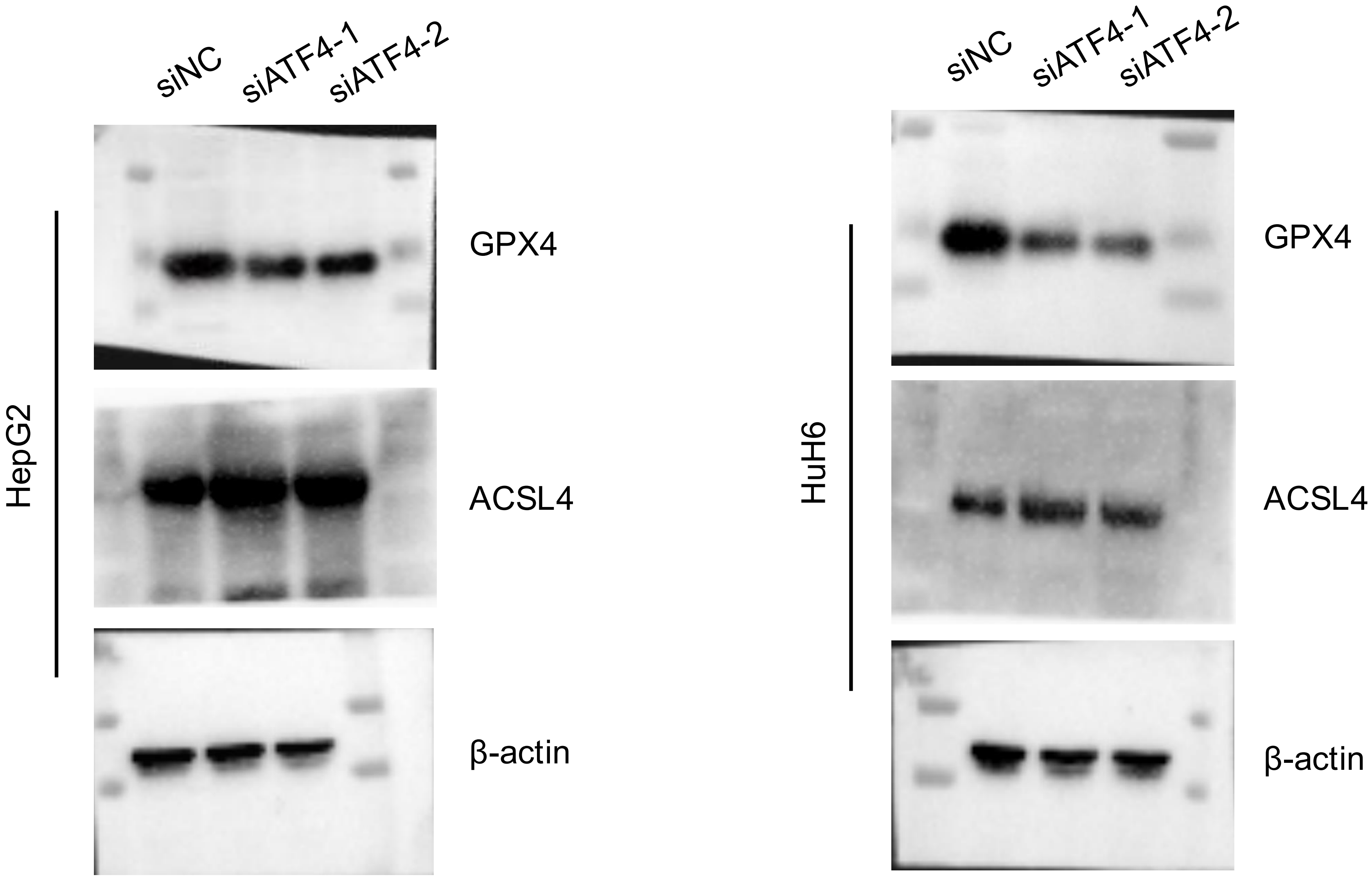
siNC
siATF4-1
siATF4-2
HepG2
GPX4
ACSL4
β-actin
siNC
siATF4-1
siATF4-2
HuH6
GPX4
ACSL4
β-actin
